# A 3D diffusional-compartmental model of the calcium dynamics in cytosol, sarcoplasmic reticulum and mitochondria of murine skeletal muscle fibers

**DOI:** 10.1371/journal.pone.0201050

**Published:** 2018-07-26

**Authors:** Lorenzo Marcucci, Marta Canato, Feliciano Protasi, Ger J. M. Stienen, Carlo Reggiani

**Affiliations:** 1 Department of Biomedical Sciences, University of Padova, Padova, Italy; 2 CeSI-Met - Center for Research on Ageing and Translational Medicine, Chieti, Italy; 3 Department of Medicine and Aging Science; University G. d’Annunzio, Chieti, Italy; 4 Department of Physiology, VU University Medical Centre, Amsterdam Cardiovascular Sciences, Amsterdam, the Netherlands; University of Debrecen, HUNGARY

## Abstract

Variations of free calcium concentration ([Ca^2+^]) are powerful intracellular signals, controlling contraction as well as metabolism in muscle cells. To fully understand the role of calcium redistribution upon excitation and contraction in skeletal muscle cells, the local [Ca^2+^] in different compartments needs to be taken into consideration. Fluorescent probes allow the determination of [Ca^2+^] in the cytosol where myofibrils are embedded, the lumen of the sarcoplasmic reticulum (SR) and the mitochondrial matrix. Previously, models have been developed describing intracellular calcium handling in skeletal and cardiac muscle cells. However, a comprehensive model describing the kinetics of the changes in free calcium concentration in these three compartments is lacking. We designed a new 3D compartmental model of the half sarcomere with radial symmetry, which accounts for diffusion of Ca^2+^ into the three compartments and simulates its dynamics at rest and at various rates of stimulation in mice skeletal muscle fibers. This model satisfactorily reproduces both the amplitude and time course of the variations of [Ca^2+^] in the three compartments in mouse fast fibers. As an illustration of the applicability of the model, we investigated the effects of Calsequestrin (CSQ) ablation. CSQ is the main Ca^2+^ buffer in the SR, localized in close proximity of its calcium release sites and near to the mitochondria. CSQ knock-out mice muscles still preserve a near-normal contractile behavior, but it is unclear whether this is caused by additional SR calcium buffering or a significant contribution of calcium entry from extracellular space, via stored-operated calcium entry (SOCE). The model enabled quantitative assessment of these two scenarios by comparison to measurements of local calcium in the cytosol, the SR and the mitochondria. In conclusion, the model represents a useful tool to investigate the impact of protein ablation and of pharmacological interventions on intracellular calcium dynamics in mice skeletal muscle.

## Introduction

The cytosolic calcium concentration ([Ca^2+^]_cyto_) plays a crucial role in the regulation of muscle contraction and relaxation [1]. In parallel with its role in regulating muscle activity, calcium also regulates ATP resynthesis in the mitochondria. Therefore, insight into the local variation in the intracellular Ca^2+^-concentration is key to understand not only the link between excitation and contraction but also its role in maintaining the balance between ATP demand and supply. The calcium-mediated regulation is critically dependent on the calcium release units (CRUs), the specialized intracellular junctions, also named triads, which are formed by the apposition of the terminal cisternae (TC) of the sarcoplasmic reticulum (SR) and the transverse tubuli (TT). The intermyofibrillar mitochondria are located near to the CRUs. Among the most important proteins composing the CRUs there are the dihydropiridine receptor (DHPR), acting as a voltage sensor, the ryanodine receptor (RyR), which is the TC calcium release channel, the SR Ca^2+^ buffer calsequestrin (CSQ), and the junctional proteins triadin and junctin. Mutations of these proteins are known to cause severe disorders and diseases, such as Malignant Hyperthermia (MH), Central Core Disease (CCD), and Vacuolar Myopathy (see for recent reviews [2,3]).

In skeletal and cardiac muscle cells, calcium ions are stored in the TC of the SR which are in close contact with the TT system, which propagates the action potential (AP) during excitation-contraction (EC) coupling. The AP triggers the release of calcium through the interaction between DHPR and RyR, so that calcium can diffuse in the cytosol. The binding of calcium to the Tn-C subunit of troponin induces a shift of the tropomyosin filament on the actin filament uncovering the actin-binding sites for myosin and allowing for the beginning of the acto-myosin cycle and force generation with the associated chemical energy (ATP) utilization. The release of calcium via RyR channels, its binding to cytosolic buffers, and the re-uptake back into SR through the sarcoplasmic endoplasmic reticulum calcium ATPases (SERCA) determines the amplitude and the time course of the cytosolic calcium ([Ca^2+^]_cyto_) transient. When the RyR channels are closed (low RyR permeability), [Ca^2+^]_cyto_ drops below the threshold required to activate the actin filaments and the muscle relaxes. Ca^2+^ uptake in the mitochondria plays an important role in modulating the resynthesis of ATP required for force generation and SR Ca^2+^ reuptake via SERCA.

Previously, models have been developed describing intracellular calcium handling in skeletal and cardiac muscle cells [4–10]. Evidence suggests that the local calcium concentrations and the calcium diffusion within the cell need to be taken into consideration [11]. Moreover, insight in the dynamics of [Ca^2+^] in all the intracellular compartments is a requirement for a full understanding of the calcium cycle triggered by APs [9]. A comprehensive model describing the kinetics of the changes in free calcium concentration in skeletal muscle fibers, and integrating the two above features, is still lacking. Therefore, we designed a 3D compartmental model which takes into account the radial symmetry of the unit cell of striated muscle (the half sarcomere) to simulate calcium dynamics at rest and during trains of stimulation at various rates in mice skeletal muscle fibers. The model includes a cytosolic compartment where myofibrils are located, a sarcoplasmic reticulum compartment to which TC are associated and a mitochondrial compartment. The experimental data for model validation are provided by determination of free calcium concentration in the cytosolic compartment [Ca^2+^]_cyto_ with ratiometric probes such as Fura 2. The determination of [Ca^2+^] in the other two most relevant intracellular compartments, the lumen of the sarcoplasmic reticulum (SR) and the mitochondrial matrix, can be achieved by more recently developed genetically targeted FRET-based fluorescent probes [12–15].

To validate our model, we first simulated the variations of free and bound [Ca^2+^] during contractile activity in the three compartments. As a further test and an illustration of the applicability of the model we investigated the effects of Calsequestrin (CSQ) ablation. In our laboratories, wild type mice and mice carrying null mutations of the genes coding for calsequestrin (CSQ-KO) have been comparatively studied for several years [12,13,16–21]. Surprisingly, CSQ knock-out mice muscles still preserve a near-normal contractile behavior, but it is unclear whether this is caused by calcium storage in additional SR Ca^2+^ buffers or by a significant contribution of Ca^2+^ entry from extracellular space, when the SR is depleted of Ca^2+^, via stored-operated Ca^2+^ entry (SOCE). The model enabled quantitative assessment of these two scenarios by comparison with measurements of local calcium in the cytosol, the SR and the mitochondria.

## Material and methods

### Modelling Ca^2+^ movements

**Fig 1:**
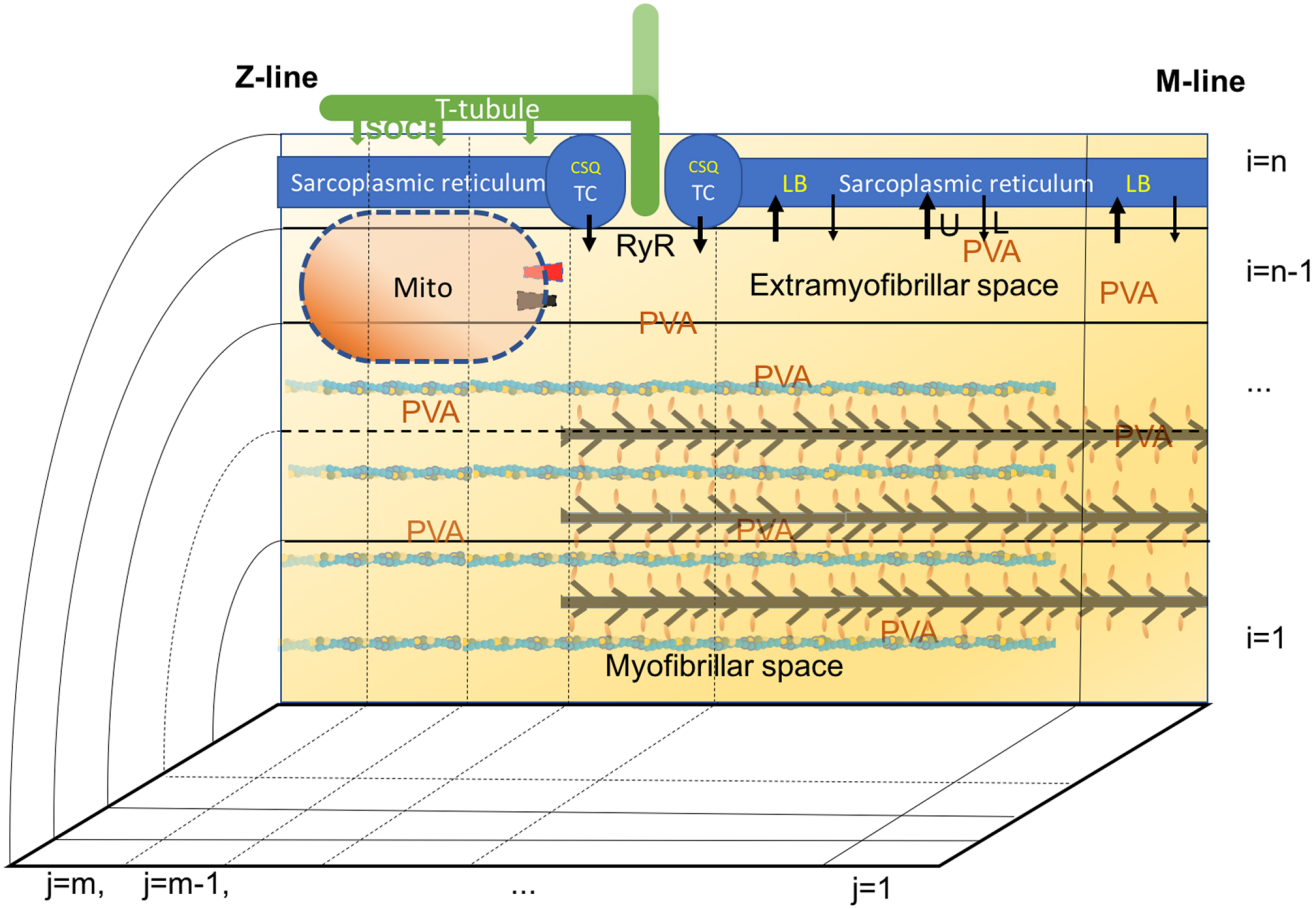
Schematic representation of the 3D compartmental diffusional model of murine skeletal muscle. The unit cell of the skeletal muscle fiber, the half sarcomere, is approximated as a cylinder, so that rotational symmetry allows for analysis in 2D space. This space is occupied by three main compartments: sarcoplasmic reticulum, cytosolic space and mitochondrion. The sarcoplasmic reticulum is divided into m longitudinal sub-compartments and the cytosolic space is divided into m longitudinal and n-1 radial sub-compartments. Diffusion of Ca^2+^ ions is analyzed between sub-compartments. The mitochondrial compartment is close, but external, to extramyofibrillar space (EF). The mitochondrial calcium uniporter (MCU in black) and sodium calcium exchanger (NCE in red) sense the [Ca^2+^] in the sub-compartment 125 nm away from the RyR, toward Z-line. Several buffers are present in distinct sub-compartments: calsequestrin (CSQ) and additional buffers (AB) in sarcoplasmic reticulum, parvalbumin (PVA) in all cytosolic spaces, troponin in the myofibrillar spaces, and a mitochondrial buffer (B) in the mitochondrial matrix. Store operated Ca^2+^ entry (SOCE) is depicted by the vertical arrows from the T-tubular network extended toward the Z-line (green).

Skeletal muscle cells are composed of a parallel arrangement of thin myofibrils of approximately 1 μm in diameter, consisting of 2.5 μm long sarcomeres surrounded by a sarcoplasmic reticular network and mitochondria. The half sarcomere can be considered as the unit cell with cylindrical geometry, from M-line to Z-line, with a radius R = 0.5 μm and a length L = 1.25 μm (values are summarized in S1 Table), with three compartments: 1. the SR in the most outer region, with a TC at the end of the thick filaments, 2. the cytosolic space where myofibrils are located and 3. the mitochondrion, in the cytosolic space near the TC (see Fig 1). The contribution of subsarcolemmal and perinuclear mitochondria to intracellular Ca^2+^ handling is assumed to be negligible in this configuration.

The SR occupies 9% of the total volume (V_tot_) including the TC, which accounts for 3.5% of the total volume V_tot_ [22]. The cytosolic space equals the sum of myofibrillar space (MS, 80% of V_tot_), which contains the myofibrils, and extramyofibrillar space (EF, 6% of V_tot_), closer to SR. The remaining 5% of V_tot_ is occupied by mitochondria [13,23,24].

Ca^2+^ fluxes between the compartments are controlled by Ca^2+^-pumps or channels, but Ca^2+^ can also diffuse within the SR and cytosolic space with a free diffusion coefficient (D) of 300 μm^2^s^-1^ [7]. The half-sarcomere is divided into m = 10 longitudinal (1 being adjacent to M-line, see Fig 1) and n = 5 radial (1 being the center of muscle cell) sub-compartments. Myofibrils occupy radial compartments from 1 to 3, the 4^th^ being the EF, and are surrounded by the SR. TC, where Ca^2+^ release takes place, are located in the 7^th^ longitudinal compartment of the 5^th^ radial sub-compartment, at a distance of approximatively 0.8 μm from the M-line. SERCA is located along all the SR compartments. Mitochondrial influx and efflux are based on the local [Ca^2+^]_cyto_ in the 8^th^ longitudinal and 4^th^ radial sub-compartment, 125 nm away from RyR. The distribution of these elements has been chosen to closely match the observed average position of mitochondria at about 130 nm distance from the TC [11,25]. The results obtained for Ca^2+^ signal in frog skeletal muscle from an 18- and a 100-compartments model have been reported to be close each other [26]. Similarly, we have compared the results for our 50-compartments model, to a finer model with m = 20 and n = 15 (300 compartments). In the finer model, average cytosolic calcium decreases by only 3%, while oscillations in the compartment with the MCU and NCE (125 nm away from the RyR), maximum [Ca^2+^] increases by 9% (from 870 to 950 nM), while the minimum is almost stable at 240 nM (see S1 Fig). Similar differences are observed also in the free [Ca^2+^]_mito_, however, the difference in the average values is less than 0.1%. Therefore the 50-compartments model is used in the simulations which also has the advantage that the computation time is suitable on a common personal computer using MatLab.

The cylindrical geometry makes the equation for the rate of the local change in [Ca^2+^] [27]:

$$\frac{\partial {[Ca}^{2+}]}{\partial t}=\frac{1}{r}\frac{\partial }{\partial r}[Dr\frac{\partial {[Ca}^{2+}]}{\partial r}]+\frac{\partial }{\partial x}[D\frac{\partial {[Ca}^{2+}]}{\partial x}]-F({[Ca}^{2+}],t)$$ (1)

in which x is the longitudinal coordinate and r the radial one, and F is the net binding flux. Using the discretization as proposed previously [4] this equation becomes:

$$\frac{{{[Ca}^{2+}]}_{i,j,t+\Delta t}-{{[Ca}^{2+}]}_{i,j,t}}{\Delta t}=4Dn/R^{2}[i{\left({[Ca}^{2+}\right]}_{i+1,j}-{{[Ca}^{2+}]}_{i,j})-(i-1)({{[Ca}^{2+}]}_{i,j}-{{[Ca}^{2+}]}_{i-1,j})]+D{\left(\frac{m}{L}\right)}^{2}[{{[Ca}^{2+}]}_{i,j+1}-2{{[Ca}^{2+}]}_{i,j}+{{[Ca}^{2+}]}_{i,j-1}]-F({{[Ca}^{2+}]}_{i,j},t).$$ (2)

Ca^2+^ ions can exit from SR to EF through RyR channel present in the TC, whose permeability is regulated by the action potential. Following the analysis and constraints proposed in [4], we assume that the permeability (P_t_) increases and decreases exponentially with time constants τ_on_ = 1 ms and τ_off_ = 5 ms. Then, the rate of Ca^2+^ release is:

$${\frac{d{Ca}^{2+}}{dt}|}_{RyR}=P_{max}[1-e^{-\frac{t}{\tau_{on}}}][e^{-\frac{t}{\tau_{off}}}]({\left[{Ca}^{2+}\right]}_{TC}-{\left[{Ca}^{2+}\right]}_{x})$$ (3)

where P_max_ is the maximum permeability which includes the release surface of the TC. The rate of release depends on the gradient of [Ca^2+^] between the TC and the sub-compartment of EF adjacent to TC (subscript x). Actually, the rate of calcium release depends on several factors which have been collected in the parameter "evacuability" [15,28], which includes permeability, release surface and intraluminal buffering. Following the procedure proposed previously [4] we fit the parameter P_max_ to obtain the experimentally observed averaged [Ca^2+^]_cyto_ in WT mice during the steady phase of the train of stimuli. This approach does not take calcium dependent inactivation into account. A reduction in the Ca^2+^ release flux from the first AP to the second and subsequent has been considered in previous models [7] to account for calcium dependent inactivation. In this study, we focus on the [Ca^2+^] in the three compartments during the steady phase, reached toward the end of a stimulation train. Therefore, we decided not to include such a phenomenon. The impact of calcium dependent inactivation, as described previously (27), in the present model is illustrated and discussed in the S1 File.

SERCA activity is modeled by simplified first-order kinetics proposed by [4], as follows:

$${\frac{d{Ca}^{2+}}{dt}|}_{Up}=\frac{V_{max}{\left[{Ca}^{2+}\right]}_{EFx}}{{\left[{Ca}^{2+}\right]}_{EFx}+K_{m}}$$ (4)

with a maximum pump rate V_max_ = 4 mmol s^-1^ per liter of fiber volume and a Ca^2+^ concentration at which half-maximum pump rate is reached, K_m_ = 0.5 μM [5,29]. This implies that SERCA is active even in non-excited cells at rest. Therefore, a constant leak term in SERCA is included to keep [Ca^2+^]_cyto_ in quiescent cells constant.

This compartmental diffusional model extends the model proposed by Cannel and Allen [4] by including a more detailed geometry. In their model, as in other diffusional models [6–8,30,31], validation was only based on cytosolic Ca^2+^ transients. Our model validation is based on experimental data of free calcium concentration in SR and mitochondria, i.e. [Ca^2+^]_SR_, and [Ca^2+^]_mito_, respectively. In this comprehensive analysis, the diffusional approach which takes into account microdomains in the space adjacent to mitocondria or SR, becomes very important. This feature is absent in compartmental models which only consider averaged concentration within compartments.

The study of calcium dynamics in the mitochondrion is a relatively new subject [9,10,24,32–34]. However, a quantitative model of skeletal muscle fibers including mitochondria is still lacking. The governing equation for total Ca^2+^ concentration in the mitochondrion [Ca^2+^]_mito,tot_ is:

$$\frac{\partial {\left[{Ca}^{2+}\right]}_{mito,tot}}{\partial t}=J_{MCU}({\left[{Ca}^{2+}\right]}_{cyto})-J_{NCE}({\left[{Ca}^{2+}\right]}_{mito},{\left[{Ca}^{2+}\right]}_{cyto})$$ (5)

J_MCU_ is the influx via the mitochondrial Ca^2+^ uniporter (MCU) and J_NCE_ is the efflux through mitochondrial Na^+^/Ca^2+^ exchanger (NCE). A detailed description of fluxes of Ca^2+^ ions through MCU, and NCE would require the analysis of all the components which influence the mitochondria membrane potential ΔΨ_m_ which in turn affects MCU influx [35]. This would imply the analysis of [H^+^], [Na^+^], [K^+^], their flux through the inner mitochondrial membrane and relative buffers in the matrix, and would introduce several additional parameters to the model. We consider such a detailed description beyond the scope of the present work, and adopt a simpler model based on a constant [Na^+^] and a constant ΔΨ_m_, which has proven to be suitable for cardiac myocytes, at relatively low frequency of stimulations [10]. In this approximation, J_MCU_ is simplified as a saturable first-order transporter independent of the internal [Ca^2+^]_mito_:

$${\text{J}}_{\text{MCU}}={\text{J}}_{\text{offset}}+{\text{f}}_{\text{MCU}}{\text{V}}_{\text{MCU}}\frac{{\left[{\text{Ca}}^{2+}\right]}_{\text{x}}{}^{\text{h}}}{{\left[{\text{Ca}}^{2+}\right]}_{\text{x}}{}^{\text{h}}+{\text{K}}_{\text{d}}{}^{\text{h}}}$$ (6)

where V_MCU_ is the maximum flux rate, [Ca^2+^]_x_ the Ca^2+^ concentration in the sub-compartments of EF where the mitochondrion resides and K_d_ the Ca^2+^ concentration where the pump rate is half-maximal. f_MCU_ is a multiplication factor to fit the data, h the Hill parameter and J_offset_ the constant flux which equilibrates the basal flux via NCE, J_NCE_.

J_NCE_ is based on a 3:1 stoichiometry for Na^+^ and Ca^2+^ [33], described with more detail in the S2 File, and contains a multiplicative factor f_NCE_ with the same role as f_MCU_. The equation used depends on the inner membrane potential ΔΨ_m_, the cytosolic and intra-mitochondrial [Ca^2+^] as well as cytosolic and intra-mitochondrial [Na^+^]. This relationship leads to symmetrical uptake and decay transients in [Ca^2+^]_mito_. However, experimental data show that [Ca^2+^]_mito_ exhibits an average rise time during train of stimuli of 27.8 ms, much faster than its exponential decay after the end of stimulation (rate constant of 250 ms^-1^). To account for this asymmetry, we propose a phenomenological modification of J_NCE_, which follows the original description proposed in [33] up to [Ca^2+^]_mito_ = 1 μM, and increases exponentially above this value (see S2 File for an extended analysis and description).

### Ca^2+^ buffers

The model includes the following Ca^2+^ buffers: Calsequestrin (CSQ) in the TC, an additional buffer (AB) in the SR, Parvalbumin (PVA) in cytosolic space (both EF and MS), Troponin C (Tn-C) in MS, and a buffer in the mitochondrial matrix (B). PVA-sites can be occupied also by Mg^2+^ whose concentration is assumed to be constant. Values for buffering capacity and binding and unbinding rates (reported in S1 Table) are assumed as in [5] for PVA (for Ca^2+^ and Mg^2+^), and [7] for Tn-C (referring to fast-twitch mouse muscle), while tentative parameters for the mitochondrial buffer are taken from [10]. The cytosolic and mitochondrial buffers are considered to be uniformly distributed into the above-mentioned spaces and no cooperativity is assumed in the Ca^2+^-binding process. Therefore, the governing equation is:

$$\frac{d{[SCa}^{2+}]}{dt}=k_{on}[{Ca}^{2+}][S]-k_{off}{[SCa}^{2+}]$$ (7)

where S and SCa^2+^ represent the free and calcium-bound form of the buffer, respectively. The previous equation should be applied to every sub-compartment in the cytosolic space, for every buffer S. However, the relative characteristic times for diffusion and binding events allow for a simplification which maintains the computation time suitable for a personal computer. The characteristic length for the diffusional event is about 1 μm, so using D = 300μm^2^s^-1^ the characteristic time is τ_D_ = 3.3 ms. The calcium binding and release from troponin are the faster ones, because calcium binding to PVA is limited by the speed of Mg^2+^ release. However, in the presence of the highest [Ca^2+^]_cyto_ = 0.38 μM considered in this study, this event has a characteristic time of about τ_B_ = 30 ms, one order of magnitude higher than τ_D_. We therefore introduced the simplification that, in each time step, the equilibrium between each buffer and the free calcium concentration is governed by the mean value of [Ca^2+^] in the cytosolic space <[Ca^2+^]_cyto_> (see also [36]). In this case, the amount of calcium bound to or released from the buffer S in each sub-compartment x, is proportional to the ratio [Ca^2+^]_x_/<[Ca^2+^]_cyto_> and, only three PDEs, one for each cytosolic buffer, describe the binding kinetics.

The concentration of CSQ isoform 1 (CSQ1), the predominant form in rodent fast-twitch skeletal muscle, is equal to about 36 μmoles per liter of total fiber volume in rat muscle fibers with a binding capacity of up to 80 mol Ca^2+^ per mole of CSQ1 [37]. In the geometry used, corresponding to murine fibers, this leads to local concentration of 82.8 mM in TC. CSQ properties are modified from [4] to account for its cooperative buffering properties. A Hill equation for cooperative binding with a Hill coefficient of 3 was used, as determined for frog skeletal muscle [38]:

$$\frac{d{[CSQCa}^{2+}]}{dt}=k_{on}{\left[{Ca}^{2+}\right]}^{3}[CSQ]-k_{off}{[CSQCa}^{2+}]$$ (8)

Eq 8 is a modified version of Eq 7 and includes the Hill coefficient as a power for the [Ca^2+^] in the right-hand-side. This introduces a reasonable approximation [39] compatible with the above-mentioned experimental data. The dissociation rate constant for CSQ was calculated from the binding rate constant in [5] and a level of 15% saturation of the total buffer capacity in quiescent fibers [40].

As to the additional buffers (AB) in the SR, a distinction has been made between linear and cooperative buffers [38]. Additional buffers are supposed to have a higher affinity but a several-fold lower capacity with respect of CSQ [41,42]. We used a Hill equation with h = 1, i.e. Eq 7, to describe the binding kinetics, with the appropriate slower rate constants. In an experimental work [38], best fitting of the experimental data was achieved using a ratio between the Ca^2+^ bound to AB and the Ca^2+^ bound to CSQ of approximately 0.1 at rest. We used this value, but also examined the effect of the maximum value of 0.2 estimated in [38].

The set of differential equations was solved using the ode15s built-in solver in MATLAB (The MathWorks Inc.). No external sources or drains are included, and the constancy of the total amount of Ca^2+^ at any time-step is used to test the reliability of the computations. Mass conservation is achieved within the limits of the software precision. Time steps are controlled by MATLAB; the validity of the results was tested using different values of absolute and relative tolerance (RelTol and AbsTol). Accuracy was tested using different initial conditions. Sensitivity analyses have been done for all the parameters used and are reported in S2 Table. Each parameter has been both increased and decreased by a 5%, while keeping other parameters constant, and the free calcium concentration in the three compartments have been compared to the values obtained in the basic WT model. Most of the modifications lead to a difference of less than 1%, and only the calsequestrin cooperativity lead to differences of more than 5%. Notably, 5% is less than the uncertainty or variability of parameters values as observed experimentally in different studies. However, the model has been compared against the data obtained in our laboratory with the same set-up and muscle type, on three compartments and for two different cell types (WT and CSQ-KO), supporting the validity of our conclusions. A more detailed analysis for mitochondrial buffering capacity and kinetics, as well as for the buffering capacity of AB, were done as described in the text and in the S3 and S4 Files.

### Experimental data

[Ca^2+^] signals, i.e. variations in free calcium concentrations, adopted as reference for fitting, and simulation were derived from data previously published, using different ratiometric calcium probes in cytosol [16], SR [12], and mitochondria [13]. Single fibers were enzymatically dissociated from murine Flexor Digitorum Brevis muscle (FDB) and studied using an inverted fluorescence microscope at rest and during brief trains of stimulation at 1, 5, 20 and 60 Hz. Cytosolic free Ca^2+^ determination was obtained loading fibers with Fura-2 acetoxymethyl ester and using a dual-beam excitation fluorescence photometry setup. Mitochondrial and SR free calcium concentrations were calculated from the single excitation, dual emission (YFP/CFP) intensity ratio after correction for background fluorescence. In these experiments, wild type (WT) mice and mice lacking both isoforms of Calsequestrin (CSQ-KO) were transfected with genetically targeted FRET-based indicators (Cameleons [43]) directed either to the SR (D1ER) or to the mitochondria (4mtD3cpv). An analysis of the ratiometric signal-calcium concentration relationship for Fura-2 and FRET based indicators (F-[Ca^2+^] or R-[Ca^2+^] respectively) has been derived in [30] and [14], which includes also a time dependent factor dF/dt and dR/dt to estimate the kinetics of binding and unbinding of the probes. Although no detailed information is available on the on-off kinetics of the D1ER and 4mtD3cpv, the experimental evidence [12,13] strongly support the view that these probes are sufficiently fast to record changes on a ms-time scale. The theoretical treatment and model simulations in S5 File support this view. All experimental data and, therefore, all simulations refer to a temperature of 25°C.

## Results

### Parameter fitting and model validation for wild type murine fast skeletal muscle fibers

#### a) Parameter fitting at 60 Hz stimulation

The numerical values of most parameters were taken from the literature, as described in the previous section. The value of P_max_ in Eq 3 was adjusted to match the experimental [Ca^2+^]_cyto_ recorded during a stimulation train at 60 Hz; the multiplicative parameters f_MCU_ and f_NCE_ and the parameters in J_NCE_ were adjusted to match the amplitude and time course of [Ca^2+^]_mito_ at 60 Hz (see Table 1). All other model parameters used are summarized in S1 Table.

**Table 1:** Experimental [Ca^2+^] in μM for WT muscle fibers. These values are averages measured at steady state during stimulation trains at various rates (60, 20, 5, and 1 Hz).

WT	60 Hz	20 Hz	5 Hz	1Hz
Cytosol	0.38	0.26	0.15	0.10
Mitochondrion	1.07	1.13	0.63	0.45
SR	362	452	446	470

**Fig 2:**
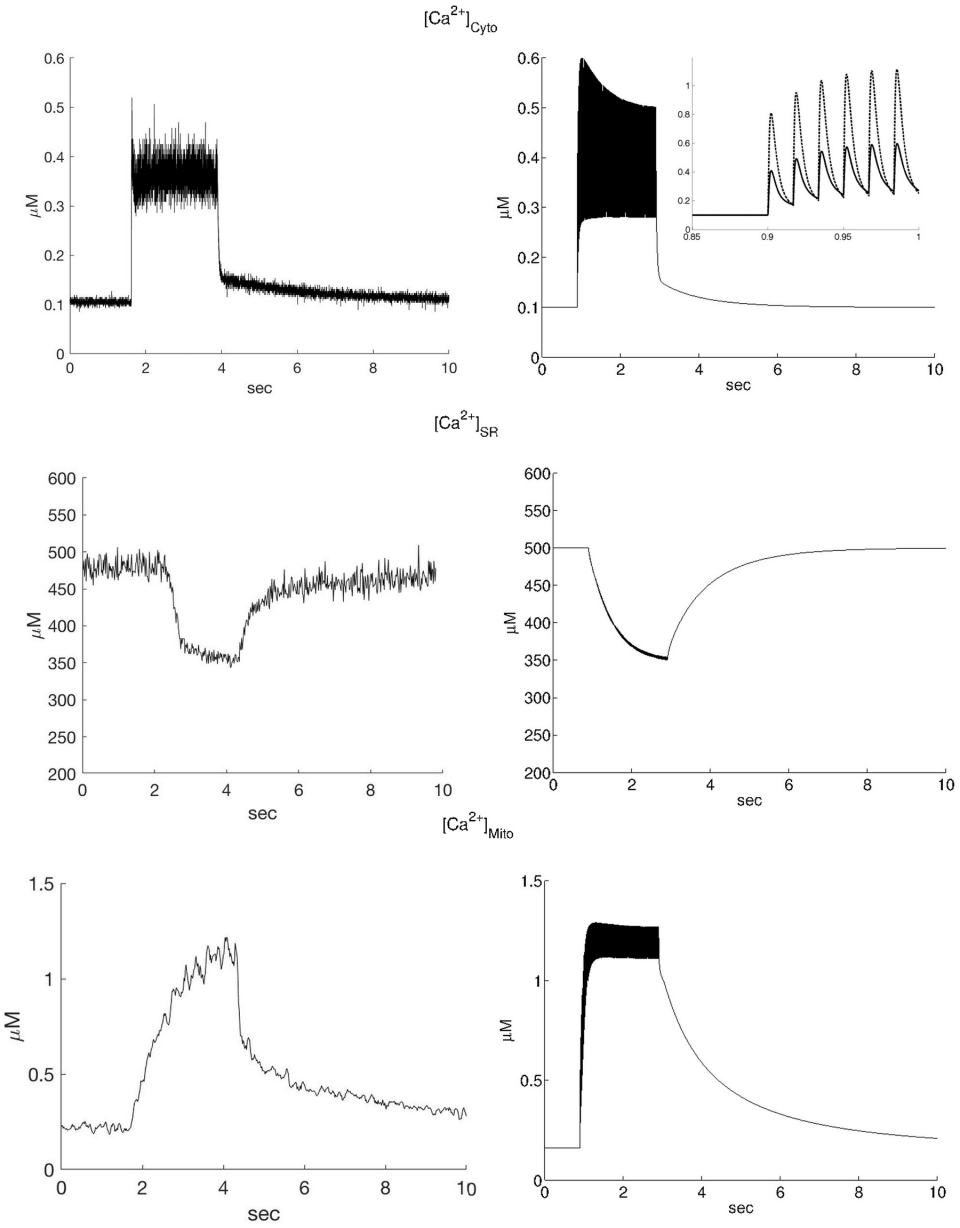
Experimental data and model simulations of calcium transients in a 2 s 60Hz stimulation train. Experimental data (left column, from [12] and [13] [Ca^2+^] calculated from Fura 2 ratio and YFP/CFP ratios according to the equations reported in S5 File) and simulations (right column) of the [Ca^2+^] transients in the cytosol (top), SR (middle) and mitochondrion (bottom) during a 2s stimulation train at 60 Hz. It can be seen that the steady-state values reached in the experiments agree well with the model simulations. In addition the time courses agree rather well considering that the model is based solely on previously published parameters. The amplitude of the oscillations in the model simulations are larger than in the experimental recordings, but this is as expected because the experimental recordings were smoothed by an x-point running average to reduce noise. In the inset of the cytosolic calcium simulation (top-right) the local Ca^2+^ concentration is shown (dotted line) in the element (microdomain) in front of MCU and NCE and compared to the average cytosolic [Ca^2+^], showing an approximately two-fold increase. The lower-right panel shows the asymmetry in the rising phase (at the beginning of the train of stimuli), and at the decay phase (at the end of the train of stimuli) in the simulated mitochondrial [Ca^2+^].

**Fig 3:**
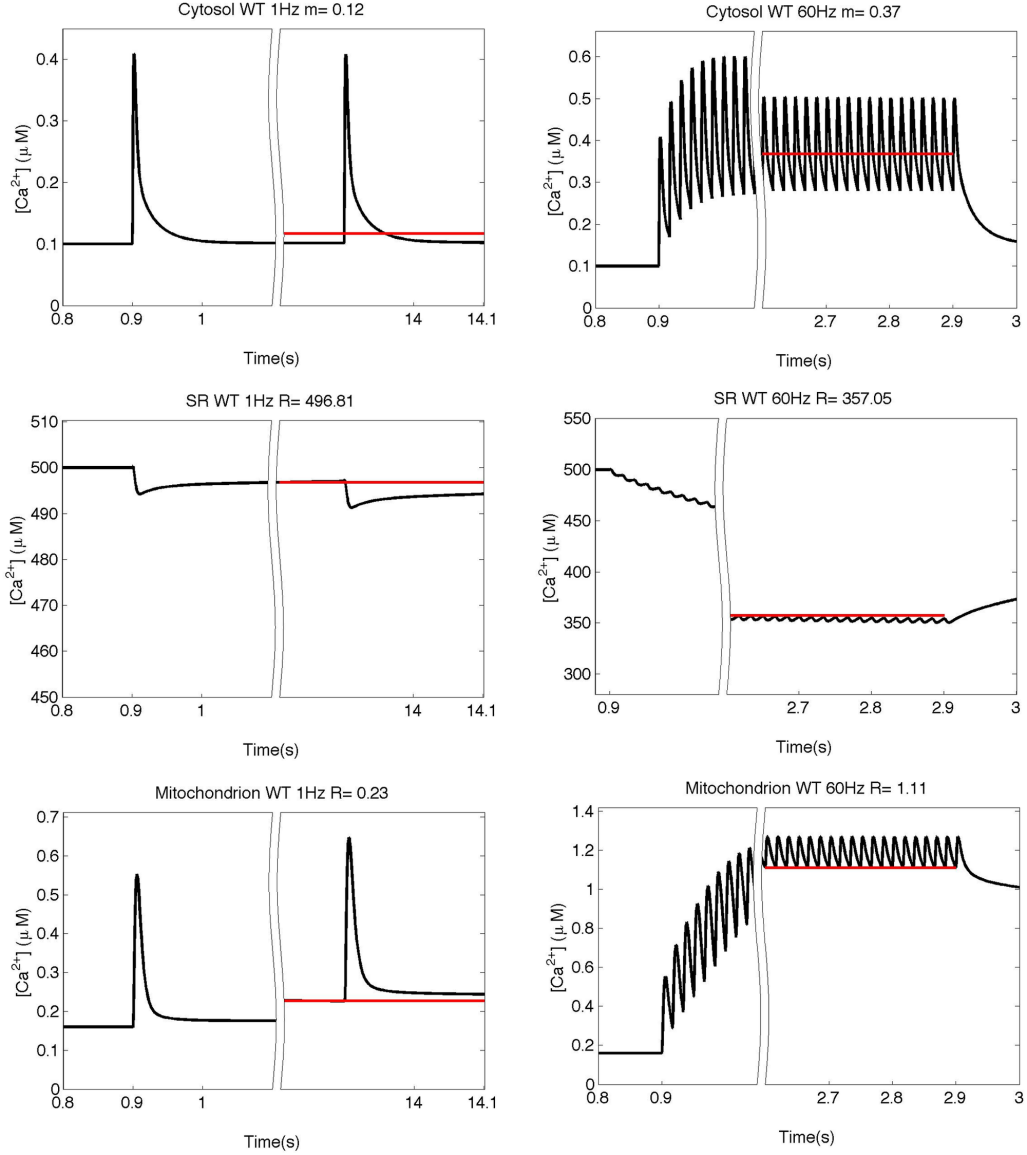
Simulation of the free calcium concentrations. Simulation of the free calcium concentrations in the three compartments at 1 and 60 Hz in the WT model at the beginning and at the end of the train of stimulation. Red lines represent the mean value (“m” for the cytosolic space) or the steady value before the beginning of the next stimulus (“s” in SR and mitochondrion). Above each panel the values for m or s are reported.

In Fig 2 a comparison is shown between the time course of the model prediction and the typical experimental data (right and left columns respectively). The values of [Ca^2+^] reported in Table 1 are extrapolated, on a relatively slow time scale (>10 ms), from the data reported in [12,13] considering the average value of the oscillations in [Ca^2+^]_cyto_ during the steady phase, as determined by measurements using Fura-2 and as proposed in [44], and the [Ca^2+^] deduced by the change in the YFP/CFP ratio for SR and mitochondrion (see red lines in Fig 3, below).

Although the model is intended primarily to simulate the final steady state values of [Ca^2+^] in the fiber compartments at 60 Hz stimulation, the kinetics of the recovery to quiescent steady state after stimulation trains are also in fair agreement with the experimental data. In particular, the asymmetry between the rising phase and the decay or recovery phase after the train of stimuli in the experimental [Ca^2+^]_mito_ (lower left panel of Fig 2) can be reproduced by the modification in J_NCE_ proposed in Methods and S2 File. Simulated recovery kinetics can be fitted by a single exponential, as in the experimental data, with rate constant t_SR_ = 0.98 s^-1^ and t_mito_ = 0.69 s^-1^, for SR and mitochondria respectively. The values are comparable to the corresponding experimental values of 0.3 s^-1^ and 0.21 s^-1^ [12,13].

The inset in the upper right panel of Fig 2 shows the transient in [Ca^2+^] in the sub-compartment of the cytosolic space where the mitochondrion is located (top figure in the right panel, dotted trace). It can be seen that the local [Ca^2+^] is more than two-fold higher than the average [Ca^2+^]_cyto_. This is in agreement with the concept of micro-domain [11] and stresses the importance of the diffusional approach followed.

#### b) Model validation by comparing the simulations at lower stimulation rates with the experimental recordings

**Fig 4:**
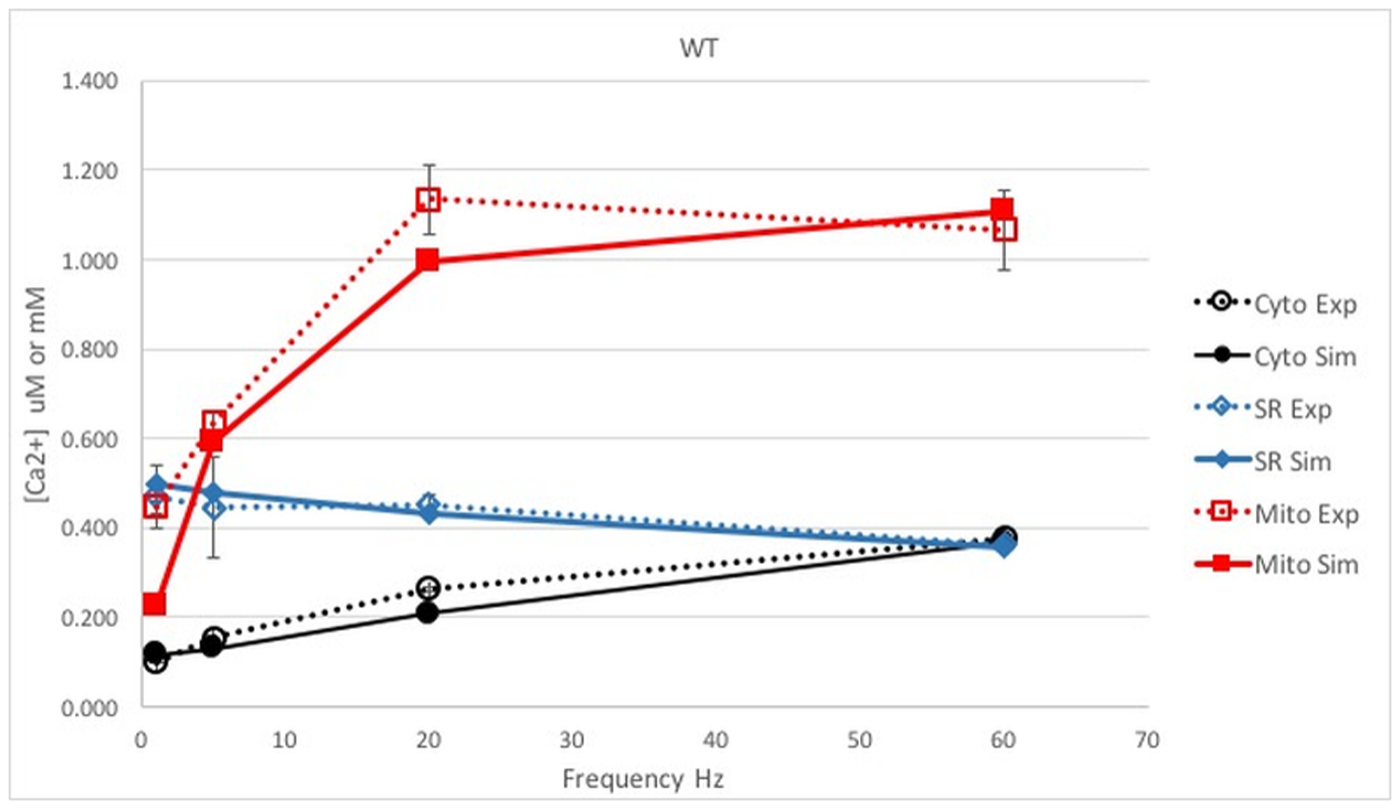
Comparison between the simulation and experiments in WT muscle fibers. The simulated [Ca^2+^]reached at steady state in the three compartments and the experimental data for 1, 5, 20 and 60 Hz in WT mouse fibers are compared with experimental values. The free model parameters are adjusted to fit the data at 60 Hz. [Ca^2+^] is reported in μM in cytosol or mitochondrion (black and red lines, respectively) or in mM for SR (blue line). Dotted lines: experimental data; continuous lines: model predictions.

The model with the parameters derived at 60 Hz stimulation was validated by considering the predictions of free calcium variations during stimulation at lower frequencies (1, 5 and 20 Hz). In Fig 4, the recorded and the predicted values are compared. The predicted values refer to the steady state condition reached at the end of the stimulation train and are denoted by the red lines in Fig 3. It can be seen that the steady state [Ca^2+^] values in all three compartments are in fair agreement with the experimental data at all stimulation rates. Simulated traces for [Ca^2+^] transients in the three compartments (cytosolic, mitochondrion and sarcoplasmic reticulum) at 1, 5 and 20 Hz stimulation and the analysis of the kinetics are reported in S3 and S6 Files.

### Model prediction for muscle fibers of CSQ-KO mice

Next, given the long experience of our laboratories with mice carrying a null mutation of CSQ [12,13,45] we tested the ability of the model to predict the impact of ablation of CSQ without any other adaptive changes, except for the permeability parameter P_max_, which was doubled to account for the increased number of RyRs, reported in [16]. Calcium can be bound by CSQ at the ratio 80 moles of calcium per 1 mole of CSQ [37], thus CSQ removal is expected to cause a marked reduction in calcium inside the SR [16], see Table 2. At high stimulation frequency, a reduction in calcium in the cytosol becomes evident; the free calcium concentration decreases after an initial peak since calcium is progressively taken up by PVA [12]. However, the drop in [Ca^2+^] is not as high as expected since the force generated is at least sufficient for normal mobility of sedentary mice.

**Table 2:** Experimental average [Ca^2+^] in μM for CSQ-KO mouse muscle fibers measured in the steady state attained during stimulation trains at various rates (1, 5, 20, 60 Hz).

		60 Hz	20 Hz	5 Hz	1Hz
CSQ-KO	Cyto (μM)	0.18	0.18	0.14	0.10
	Mito (μM)	0.74	0.73	0.62	0.43
	SR (μM)	104	177	302	440

**Fig 5:**
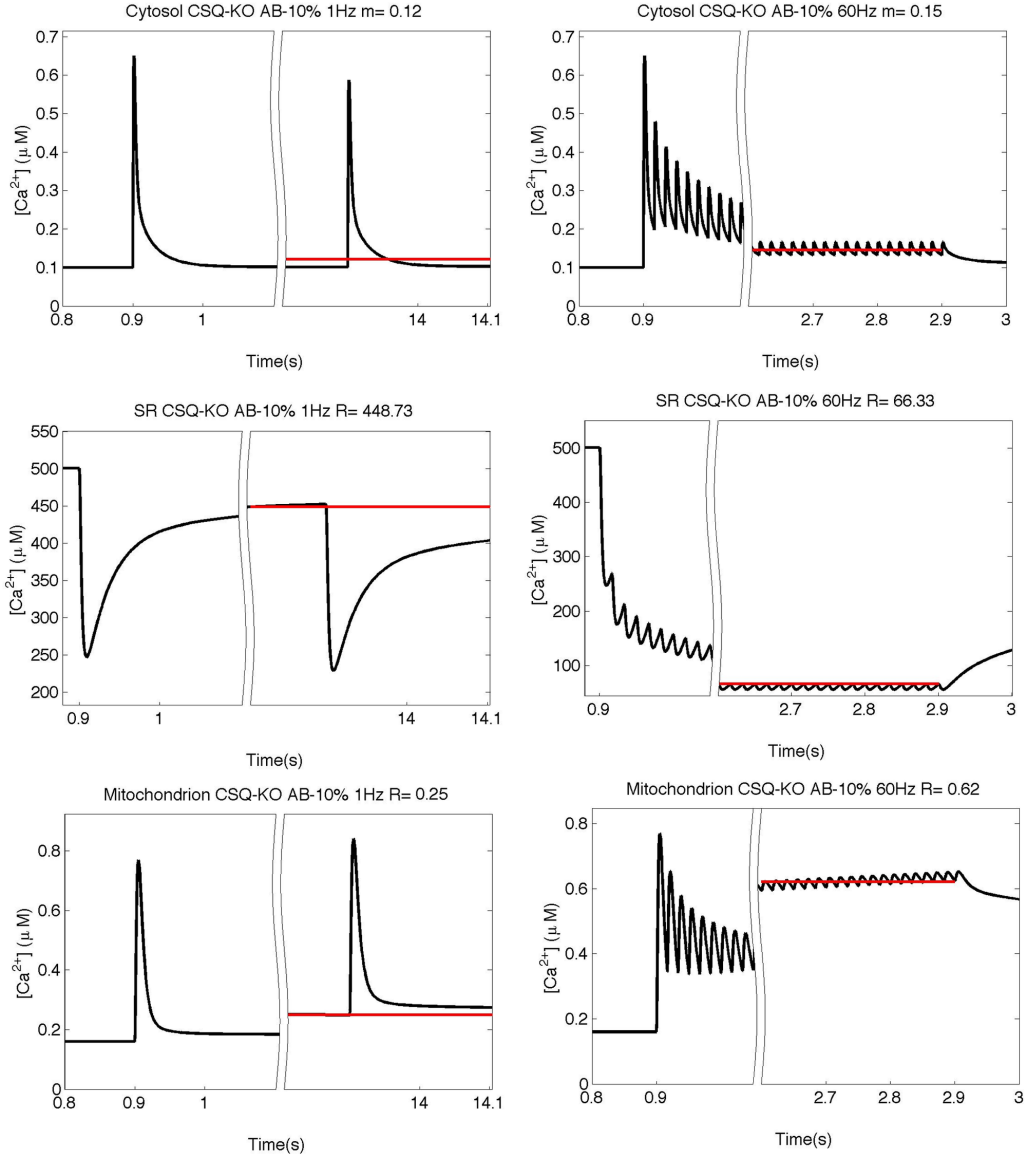
Simulation of the free calcium concentrations in CSQ-KO with an AB buffering capacity of 10% of that of CSQ. Simulation of the free calcium concentrations in the three compartments at 1 and 60 Hz as predicted by the model without CSQ (CSQ-KO) with a AB buffering capacity of 10% of that of CSQ and with doubled P_max_ value. Red lines represent the mean value (“m” for the cytosolic space) or the steady value before the beginning of the next stimulus (“s” in SR and mitochondrion). Above each panel the values for m or s are reported.

**Fig 6:**
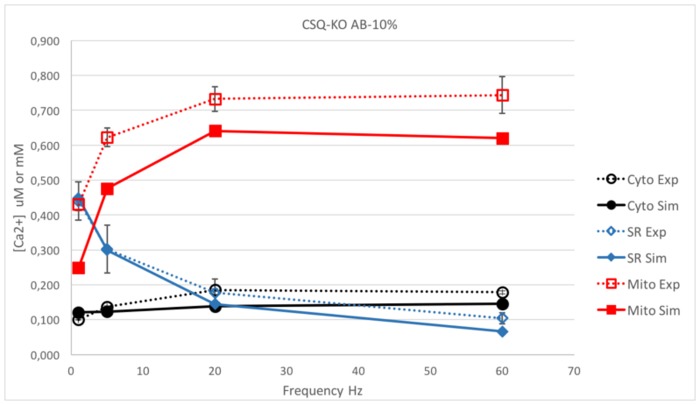
Comparison between the simulation and experiments in CSQ-KO with an AB buffering capacity of 10% of that of CSQ. Comparison between the simulated steady state [Ca^2+^] in the three compartments and the experimental data for 1, 5, 20 and 60 Hz in CSQ-KO mouse with 10% of total calcium bound in the SR at rest accounted by AB and with doubled Pmax value. [Ca^2+^] is reported in μM in cytosol or mitochondrion (black and red lines, respectively) or in mM for SR (blue line). Dotted lines stand for experimental data, continuous lines for simulations. The model without CSQ (CSQ-KO), with 10% of AB capacity and double Pmax fails to match the observed values in all three compartments, with predicted values lower than experimental data.

The mechanisms behind these near-normal contractions are not well understood. The presence of other calcium buffering proteins in the SR, such as sarcalumenin, calreticulin, calnexin, junctate and others [42,46–49], could partially alleviate calcium shortage [50]. Without additional buffers, the model predicted a marked drop in [Ca^2+^] in all three compartments due to ablation of CSQ (data not shown). Results of the model with an additional buffer (AB) with a buffer capacity ratio relative to CSQ of 0.1 are reported in Fig 5 for the response to stimulation trains at 1 and 60 Hz, and are compared with experimental data in Fig 6 for the whole range of stimulation frequencies (1–60 Hz). As can be seen in Fig 5, the simulated [Ca^2+^]_cyto_ has an initial peak similar to that predicted for WT, but it rapidly decreases to a lower steady value, while the [Ca^2+^]_SR_ shows a much stronger depletion compared to WT. Qualitatively, these results resemble the behavior of free [Ca^2+^] in SR and cytosol in CSQ-KO cells [12]. Quantitatively, however, the model prediction for [Ca^2+^]_mito_ is different from what is observed experimentally as [Ca^2+^]_mito_ is not able to maintain the relatively high values observed in CSQ-KO, especially at high stimulation frequencies.

In an attempt to improve the prediction of [Ca^2+^]_mito_ in CSQ-KO, we considered that doubling of the number of RyRs might lead to an increased RyR leakage in basal conditions. Since the basal [Ca^2+^]_cyto_ is the same in CSQ-KO and WT fibers, SERCA needs to pump twice as much. This will lead to a constant average [Ca^2+^]_cyto_, but higher local concentrations near the RyR. In principle, the higher local [Ca^2+^]_cyto_ might allow for the observed higher basal [Ca^2+^]_mito_ (230 nM in CSQ-KO vs 160 nM in WT [13]) because of the proximity of the mitochondrion to the sites of release. However, the increase predicted after doubling of the number of RyRs is clearly insufficient, since it increased the basal [Ca^2+^]_mito_ by less than 1%.

**Fig 7:**
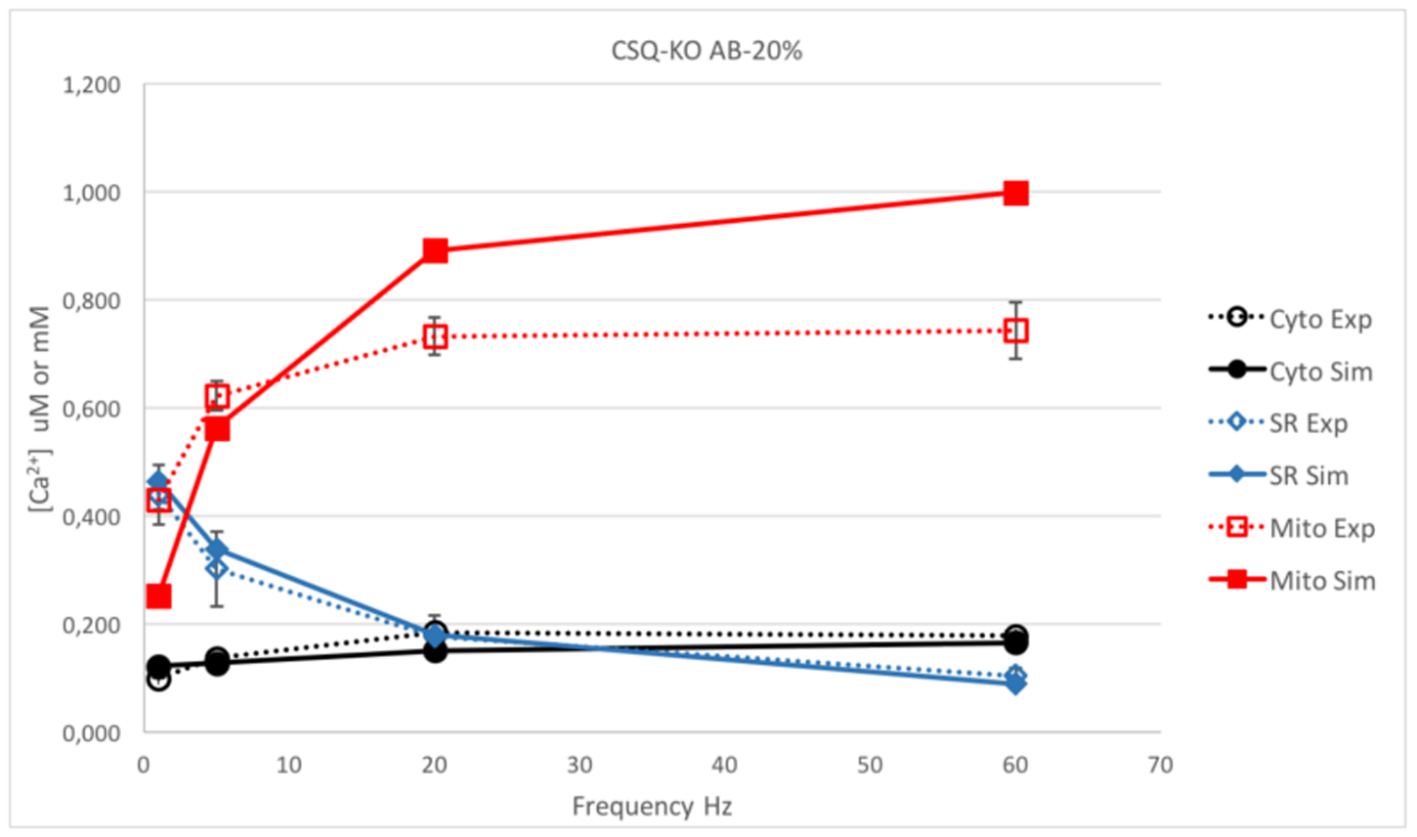
Comparison between the simulation and experiments in CSQ-KO with an AB buffering capacity of 20% of that of CSQ. Comparison between the simulated steady state [Ca^2+^] in the three compartments and the experimental data for 1, 5, 20 and 60 Hz in CSQ-KO mouse with increased AB buffering to 20% of the capacity of CSQ. [Ca^2+^] is reported in μM in cytosol or mitochondrion (black and red lines, respectively) or in mM for SR (blue line). Dotted lines stand for experimental data, continuous lines for simulations.

In a further attempt to improve the simulated calcium kinetics in CSQ-KO fibers, we considered an increase in the additional buffer capacity inside SR. An estimate of the relative amount of Ca^2+^ bound to CSQ and secondary additional buffers with linear properties has been made in frog skeletal muscles [38]. In that study, the maximum limit of Ca^2+^ bound to the secondary buffer was inferred to be 20% of total calcium bound in the sarcoplasmic reticulum at rest. In our simulations, if 20% of total SR calcium is bound to AB, [Ca^2+^]_cyto_ at 60 Hz is close to the experimental data in CSQ-KO (see Fig 7). However, Table 3 illustrates that the predicted values of free calcium in the SR ([Ca^2+^]_SR_) during 60 Hz stimulation would be lower than those observed experimentally (predicted/experimental ratio = 0.86), indicating that even in this extreme case the effect of the AB is insufficient to sustain the near-normal contraction at high frequencies. Thus, the model suggests that an extra source of external calcium (possibly via SOCE) is needed as hypothesized in [12] and recently discussed in [45].

**Table 3:** Simulated/Experimental ratio for [Ca^2+^] in μM for WT and CSQ-KO fibers, assuming the amount bound by secondary buffer capacity at 10% or at 20%. The values are calculated at the steady state during the trains of stimulations, as in the Figs 3, 6 and 7.

		60 Hz	20 Hz	5 Hz	1Hz
WT 10%	Cyto	0.98	0.79	0.84	1.17
	Mito	1.04	0.88	0.94	0.51
	SR	0.99	0.95	1.08	1.06
CSQ-KO 10%	Cyto	0.81	0.75	0.90	1.21
	Mito	0.83	0.88	0.76	0.58
	SR	0.64	0.82	0.99	1.02
CSQ-KO 20%	Cyto	0.93	0.82	0.93	1.22
	Mito	1.34	1.22	0.90	0.59
	SR	0.86	1.02	1.12	1.06

## Discussion and conclusions

In this study, we designed a new model to reproduce the Ca^2+^ dynamics in skeletal muscle fibers of WT mice. The model is based on previously published models which account for different intracellular compartments as well as for Ca^2+^ diffusion [4,7]. Compared to previous diffusional models, this is the first to take into consideration the three main compartments (cytosol, SR and mitochondria), and the first to be validated against a comprehensive set of experimental data on Ca^2+^ concentrations measured in these three compartments in murine muscle fibers. The numerical values of the parameters adopted in this study largely correspond to literature data. When data for mouse were not available, we adopted values from other studies as initial guesses and analyzed the effects of their variability (S2 Table). This was the case for the mitochondrial buffer and additional buffer in the SR.

A strong feature, illustrating the robustness of the model, is that only few parameters have been derived by fitting the model prediction to experimental data. These are: P_max_, used to match the transient variations in [Ca^2+^]_cyto_ upon electrical stimulation; f_MCU_ and f_NCE_, as well as the parameters for the exponential increase in J_NCE_, [Ca^2+^]_th_ and γ (see S2 File), used to mimic the transients in the rise and decay phases of the [Ca^2+^]_mito_. The fitting was based on the Ca^2+^ concentrations recorded during a stimulation train at 60 Hz, and the results were validated as they provided a very good prediction for the Ca^2+^ concentration variations at lower stimulation frequencies (1, 5 and 20 Hz) in all three compartments. The model was then applied to predict the free calcium concentrations in the three compartments of muscle fibers devoid of CSQ (CSQ-KO). These results provided an additional test of the model and allowed us to estimate the impact of an additional buffer inside the SR.

The results showed that even the maximal buffer capacity of a secondary buffer (AB) accounting for 20% of the buffering power of CSQ in WT fibers [38] was insufficient to explain the data and this lends support for the hypothesis that external influx via SOCE would be required to account for the missing Ca^2+^. Interestingly, fast recruitment of SOCE has been shown in CSQ-KO myotubes [51]. A recent study [52] suggests that in skeletal muscle SOCE occurs in close spatial relation with the SR Ca^2+^-pumps, in specialized structures extending from the triad toward the Z-line, as schematically indicated in Fig 1. In this situation, free [Ca^2+^]_SR_ would be influenced by the SR buffer capacity and external Ca^2+^-influx via SOCE. As a consequence, the total Ca^2+^ within the fibers would increase and the calcium entry during one contractile cycle should benefit the subsequent one, as extrusion of the Ca^2+^ from the fibers is expected to occur more slowly than entry via SOCE.

An estimate of the extra amount of calcium needed to reproduce the experimental data in CSQ-KO fibers can be obtained as follow. In the model simulation of a short (2 s) stimulation train at 60 Hz in WT muscle fibers, approximately 260 μM of calcium (or 260 μmoles/liter fiber) move from the SR to the cytosol, 250 μM derived from CSQ and 10 μM from AB which has a higher affinity and a lower capacity compared to CSQ. When CSQ is absent, the contribution of AB in the model rises in the extreme case to c.a. 60 μM in direct relation to the lowering of the [Ca^2+^]_SR_. Such an amount is well below what is required to keep cytosolic levels comparable not only to those of WT muscle fibers (approximately 180 μM would be needed in addition) but also to those experimentally observed in CSQ muscles (a contribution in the order of 100 μM would be required). This latter value corresponds (within 2 s) with a flux of 50 μM/s which is, in view of the uncertainties in the experimental determinations and in the calculations, in fair agreement with the experimentally estimated value of SOCE of 19 μM/s in [53].

The inclusion of mitochondrial calcium handling in the model represents a novelty for skeletal muscle fibers. Compared to other cell types, the volume occupied by mitochondria in skeletal muscle fibers is relatively small (approximately 5% vs. 30% in cardiomyocytes), and its contribution to Ca^2+^ homeostasis has been generally neglected (see [7]). Their localization in close proximity to the CRUs, which allows mitochondria to exploit Ca^2+^ microdomains [11] and the presence of specific components of the MCU, as for example MICU1.1 [54] which enhances Ca^2+^ transport, opens to the possibility that the amount of Ca^2+^ taken up by the mitochondria is greater than previously predicted. In addition, being close to TC and T-tubules the mitochondria might become a reliable indicator of the amount of Ca^2+^ released by RyR channels, if properly modeled.

These considerations prompted us to introduce mitochondria in the model. However, quantitative description of the mitochondrial Ca^2+^ kinetics is not an easy task. Three processes need to be considered: uptake by MCU, buffering, and release via NCE. In the last decades, several studies have proposed models of these three processes, mostly with reference to cardiomyocytes [9,24,33,34,55]. At the same time, probes for measuring intra-mitochondrial calcium concentrations have been developed and one of them (cameleon 4mtD3cpv) has been used to obtain the data analyzed in this study [13]. Considering that the aim of the present study was to provide an overall global quantitative simulation of Ca^2+^ dynamics in skeletal muscle fibers, several simplifications of the three processes (uptake-buffering-release) were introduced. Such simplifications proved to be suitable to simulate free calcium variations in the mitochondrial matrix, at rest and during trains of stimuli, in WT and CSQ-KO fibers. Our model of mitochondria was able to account for a number of observed behaviors, including the asymmetry of mitochondrial Ca^2+^ uptake and release. The emerging picture is that mitochondria take up a minor amount of calcium, thus confirming previous notions discussed in [13].

It is important to recall that the model has several limitations:

1. The model does not include the Ca^2+^-dependent inactivation (CDI), i.e. the reduction in RyR permeability during a train of APs due to the increased Ca^2+^ concentration on the cytosolic side of RyR [7]. However, as shown in S1 File, we tested the CDI effect adopting an exponential decay of P_max_ in accordance to [7]. The impact of such variation in permeability is well visible in the first stimuli of a train, but becomes rather small in the stimulation trains lasting for 1 or more seconds.
2. It is known that SERCA activity is cooperative and models are available to account for this, for example [5,56]. For the sake of simplicity we followed the proposal (see [4]) that the behavior of the pump could be simulated by Michelis-Menten kinetics, paying due attention to the choice of the values of K_m_ and V_max_.
3. The simulation of the calcium uptake via MCU and release via NCE could be improved by taking the influence of Na^+^ and H^+^ homeostasis and ΔΨ_m_ into account, as suggested by our modification of the dependency of J_NCE_ on [Ca^2+^]_mito_. These aspects warrant further study because quantitative data on mitochondria in living skeletal muscle fibers are still scarce.

In conclusion, the present model is implemented on a robust background dating back 30 years ago [4,6], but represents a significant improvement as it is the first to take into account all three major compartments, cytosol, SR lumen and mitochondrial matrix, and is validated by experimental data on calcium dynamics at rest and during trains of stimuli in all three compartments in skeletal muscle. The model has been tested in its ability to predict the calcium dynamics in CSQ-KO muscle fibers. The choice of this particular transgenic model was inspired by the specific experience of our laboratories [12,13,16,45], but also by the special relevance of this protein in the derailment of calcium kinetics in muscle disorders. Beside these specific results, the test on CSQ-KO muscle fibers illustrates the predictive ability of the model and its power to interpret and predict experimental results. Our model thus has the potential to be a helpful tool in the quantitative analyses of alterations of calcium dynamics in muscle fibers of genetically modified mice strains and to further study the impact of interventions, such as protein ablations or pharmacological treatments, on intracellular calcium dynamics.

## Supporting information

S1 Table Parameters definitions and values. (PDF)

S2 TableSensitivity analysis.To evaluate the sensitivity of the model predictions on the actual model parameters, a change of ±5% has been applied to each parameter reported in Table 2, relative to the value given in Table 1. The variations of free [Ca^2+^] in the three compartments (cytosol, SR, mitochondrion) using these modified parameters ([Ca^2+^]±5%) relative to the values obtained with the parameter values in Table 1 ([Ca^2+^]basic) are calculated and expressed as a percentage (-5%/+5%). All percentages are lower than 5% except for the Hill parameter (an exponential term) in the calsequestrin equilibrium equation. The values of $100∙(\frac{{\left[{Ca}^{2+}\right]}_{5\%}}{{\left[{Ca}^{2+}\right]}_{basic}}-1)$ in the three compartments are reported. “-”means less than 0.1% deviation. Values are given for the -5%/+5% cases.(PDF)

S1 Fig Effect of the compartment dimension. Simulation of the [Ca^2+^] in the cytosol where NCE and MCU are supposed to be located (125 nm away from the RyR) and in the mitochondrion using the 50-compartments model (red) and a 300-compartments model (blue).
(PDF)

S1 File Ca^2+^-dependent inactivation of SR Ca^2+^-release. Effect of the Ca^2+^ dependent inactivation on the model prediction.
(PDF)

S2 File Mitochondrion modeling. Equations for the MCU and NCE fluxes in mitochondrion and phenomenological hypothesis.
(PDF)

S3 File Analysis of the kinetics of the decay phase of the transients in sarcoplasmic reticulum and mitochondrion. Although the model was designed mainly to simulate the steady state concentrations reached at rest or during stimulation trains in the three main compartments, also the kinetics after the train of stimuli were well simulated, both in SR and in mitochondrion.
(PDF)

S4 File Sensitivity analysis of the mitochondrial buffer parameters. The nature of the mitochondrial buffer is largely unknown at present, and experimental data needed for its mathematical characterization are not available in literature. We have analyzed the [Ca^2+^]_mito_ behavior for different choices of the buffering capacity and kinetics, keeping a ratio for free-to-bound [Ca^2+^] of 1:100.
(PDF)

S5 File Kinetics and steady state determination of Ca2+ concentrations. Effect of the kinetic term on the [Ca^2+^] in the three compartments.
(PDF)

S6 File Time course of the variations in free [Ca^2+^] for the three compartments. Time course of the variations in free [Ca^2+^] for the three compartments at different stimulation rates in WT fibers and CSQ-KO fibers with 10% and 20% of total calcium bound accounted by secondary buffer LB.
(PDF)

S1 Video Intracellular dynamics of the [Ca^2+^]_cyto_ during the steady state phase of a train of stimuli at 60 Hz frequency in the wild type model. The peak corresponds to the position of the ryanodine receptors.
(AVI)

## References

[1] Gordon AM, Homsher E, Regnier M. Regulation of Contraction in Striated Muscle. Physiol Rev. 2000;80: 853–924. 10.1152/physrev.2000.80.2.853 10747208

[2] MacLennan DH, Zvaritch E. Mechanistic models for muscle diseases and disorders originating in the sarcoplasmic reticulum. Biochim Biophys Acta BBA—Mol Cell Res. 2011;1813: 948–964. 10.1016/j.bbamcr.2010.11.009 21118704

[3] Ríos E, Figueroa L, Manno C, Kraeva N, Riazi S. The couplonopathies: A comparative approach to a class of diseases of skeletal and cardiac muscle. J Gen Physiol. 2015;145: 459–474. 10.1085/jgp.201411321 26009541 PMC4442791

[4] Cannell MB, Allen DG. Model of calcium movements during activation in the sarcomere of frog skeletal muscle. Biophys J. 1984;45: 913–925. 10.1016/S0006-3495(84)84238-1 6733242 PMC1434964

[5] Westerblad H, Allen DG. The role of sarcoplasmic reticulum in relaxation of mouse muscle; effects of 2, 5-di (tert-butyl)-1, 4-benzohydroquinone. J Physiol. 1994;474: 291 8006816 10.1113/jphysiol.1994.sp020022 PMC1160318

[6] Baylor SM, Hollingworth S. Model of Sarcomeric Ca2+ Movements, Including ATP Ca2+ Binding and Diffusion, during Activation of Frog Skeletal Muscle. J Gen Physiol. 1998;112: 297–316. 9725890 10.1085/jgp.112.3.297 PMC2229419

[7] Baylor SM, Hollingworth S. Simulation of Ca ^2+^ Movements within the Sarcomere of Fast-Twitch Mouse Fibers Stimulated by Action Potentials. J Gen Physiol. 2007;130: 283–302. 10.1085/jgp.200709827 17724162 PMC2151645

[8] Hollingworth S, Kim MM, Baylor SM. Measurement and simulation of myoplasmic calcium transients in mouse slow-twitch muscle fibres: Calcium movements in slow-twitch fibres. J Physiol. 2012;590: 575–594. 10.1113/jphysiol.2011.220780 22124146 PMC3379702

[9] Hatano A, Okada J, Washio T, Hisada T, Sugiura S. A Three-Dimensional Simulation Model of Cardiomyocyte Integrating Excitation-Contraction Coupling and Metabolism. Biophys J. 2011;101: 2601–2610. 10.1016/j.bpj.2011.10.020 22261047 PMC3297787

[10] Wüst RCI, Helmes M, Martin JL, van der Wardt TJT, Musters RJP, van der Velden J, et al Rapid frequency-dependent changes in free mitochondrial calcium concentration in rat cardiac myocytes: Mitochondrial calcium handling. J Physiol. 2017;595: 2001–2019. 10.1113/JP273589 28028811 PMC5350475

[11] Rizzuto R, Pozzan T. Microdomains of Intracellular Ca2+: Molecular Determinants and Functional Consequences. Physiol Rev. 2006;86: 369–408. 10.1152/physrev.00004.2005 16371601

[12] Canato M, Scorzeto M, Giacomello M, Protasi F, Reggiani C, Stienen GJM. Massive alterations of sarcoplasmic reticulum free calcium in skeletal muscle fibers lacking calsequestrin revealed by a genetically encoded probe. Proc Natl Acad Sci. 2010;107: 22326–22331. 10.1073/pnas.1009168108 21135222 PMC3009789

[13] Scorzeto M, Giacomello M, Toniolo L, Canato M, Blaauw B, Paolini C, et al Mitochondrial Ca2+-Handling in Fast Skeletal Muscle Fibers from Wild Type and Calsequestrin-Null Mice. Kanzaki M, editor. PLoS ONE. 2013;8: e74919 10.1371/journal.pone.0074919 24098358 PMC3789688

[14] Sztretye M, Yi J, Figueroa L, Zhou J, Royer L, Allen P, et al Measurement of RyR permeability reveals a role of calsequestrin in termination of SR Ca2+ release in skeletal muscle. J Gen Physiol. 2011;138: 231–247. 10.1085/jgp.201010592 21788611 PMC3149434

[15] Royer L, Sztretye M, Manno C, Pouvreau S, Zhou J, Knollmann BC, et al Paradoxical buffering of calcium by calsequestrin demonstrated for the calcium store of skeletal muscle. J Gen Physiol. 2010;136: 325–338. 10.1085/jgp.201010454 20713548 PMC2931149

[16] Paolini C, Quarta M, Nori A, Boncompagni S, Canato M, Volpe P, et al Reorganized stores and impaired calcium handling in skeletal muscle of mice lacking calsequestrin-1: Calsequestrin role in skeletal muscle. J Physiol. 2007;583: 767–784. 10.1113/jphysiol.2007.138024 17627988 PMC2277031

[17] Dainese M, Quarta M, Lyfenko AD, Paolini C, Canato M, Reggiani C, et al Anesthetic- and heat-induced sudden death in calsequestrin-1-knockout mice. FASEB J. 2009;23: 1710–1720. 10.1096/fj.08-121335 19237502 PMC2698659

[18] Protasi F, Paolini C, Dainese M. Calsequestrin-1: a new candidate gene for malignant hyperthermia and exertional/environmental heat stroke. J Physiol. 2009;587: 3095–3100. 10.1113/jphysiol.2009.171967 19417098 PMC2727019

[19] Paolini C, Quarta M, D’Onofrio L, Reggiani C, Protasi F. Differential effect of calsequestrin ablation on structure and function of fast and slow skeletal muscle fibers. J Biomed Biotechnol. 2011;2011: 634075 10.1155/2011/634075 21941434 PMC3173739

[20] Protasi F, Paolini C, Canato M, Reggiani C, Quarta M. Lessons from calsequestrin-1 ablation in vivo: much more than a Ca(2+) buffer after all. J Muscle Res Cell Motil. 2011;32: 257–270. 10.1007/s10974-011-9277-2 22130610

[21] Tomasi M, Canato M, Paolini C, Dainese M, Reggiani C, Volpe P, et al Calsequestrin (CASQ1) rescues function and structure of calcium release units in skeletal muscles of CASQ1-null mice. AJP Cell Physiol. 2012;302: C575–C586. 10.1152/ajpcell.00119.2011 22049211

[22] Mobley BA, Eisenberg BR. Sizes of components in frog skeletal muscle measured by methods of stereology. J Gen Physiol. 1975;66: 31–45. 1159401 10.1085/jgp.66.1.31 PMC2226188

[23] Luff AR, Atwood HL. Changes in the sarcoplasmic reticulum and transverse tubular system of fast and slow skeletal muscles of the mouse during postnatal development. J Cell Biol. 1971;51: 369–383. 5112650 10.1083/jcb.51.2.369 PMC2108137

[24] Williams GSB, Boyman L, Chikando AC, Khairallah RJ, Lederer WJ. Mitochondrial calcium uptake. Proc Natl Acad Sci. 2013;110: 10479–10486. 10.1073/pnas.1300410110 23759742 PMC3696793

[25] Boncompagni S, Rossi AE, Micaroni M, Beznoussenko GV, Polishchuk RS, Dirksen RT, et al Mitochondria are linked to calcium stores in striated muscle by developmentally regulated tethering structures. Mol Biol Cell. 2009;20: 1058–1067. 10.1091/mbc.E08-07-0783 19037102 PMC2633377

[26] Hollingworth S, Soeller C, Baylor SM, Cannell MB. Sarcomeric Ca2+ gradients during activation of frog skeletal muscle fibres imaged with confocal and two-photon microscopy. J Physiol. 2000;526: 551–560. 10.1111/j.1469-7793.2000.t01-1-00551.x 10922007 PMC2270039

[27] Crank J. The Mathematics of Diffusion. Clarendon Press; 1979.

[28] Royer L, Pouvreau S, Ríos E. Evolution and modulation of intracellular calcium release during long-lasting, depleting depolarization in mouse muscle: Calcium flux and depletion in muscle. J Physiol. 2008;586: 4609–4629. 10.1113/jphysiol.2008.157990 18687715 PMC2614033

[29] Lytton J, Westlin M, Burk SE, Shull GE, MacLennan DH. Functional comparisons between isoforms of the sarcoplasmic or endoplasmic reticulum family of calcium pumps. J Biol Chem. 1992;267: 14483–14489. 1385815

[30] Jiang Y-H, Klein MG, Schneider MF. Numerical Simulation of Ca2+?Sparks? in Skeletal Muscle. Biophys J. 1999;77: 2333–2357. 10.1016/S0006-3495(99)77072-4 10545338 PMC1300512

[31] Novo D, DiFranco M, Vergara JL. Comparison between the Predictions of Diffusion-Reaction Models and Localized Ca2+ Transients in Amphibian Skeletal Muscle Fibers. Biophys J. 2003;85: 1080–1097. 10.1016/S0006-3495(03)74546-9 12885654 PMC1303228

[32] Cortassa S, Aon MA, O’Rourke B, Jacques R, Tseng H-J, Marbán E, et al A Computational Model Integrating Electrophysiology, Contraction, and Mitochondrial Bioenergetics in the Ventricular Myocyte. Biophys J. 2006;91: 1564–1589. 10.1529/biophysj.105.076174 16679365 PMC1518641

[33] Dash RK, Beard DA. Analysis of cardiac mitochondrial Na ^+^ -Ca ^2+^ exchanger kinetics with a biophysical model of mitochondrial Ca ^2+^ handing suggests a 3: 1 stoichiometry: Characterization of mitochondrial NCE stoichiometry. J Physiol. 2008;586: 3267–3285. 10.1113/jphysiol.2008.151977 18467367 PMC2538784

[34] Boyman L, Chikando AC, Williams GSB, Khairallah RJ, Kettlewell S, Ward CW, et al Calcium Movement in Cardiac Mitochondria. Biophys J. 2014;107: 1289–1301. 10.1016/j.bpj.2014.07.045 25229137 PMC4167535

[35] Gunter TE, Pfeiffer DR. Mechanisms by which mitochondria transport calcium. Am J Physiol-Cell Physiol. 1990;258: C755–C786. 10.1152/ajpcell.1990.258.5.C755 2185657

[36] Wagner J, Keizer J. Effects of rapid buffers on Ca2+ diffusion and Ca2+ oscillations. Biophys J. 1994;67: 447–456. 10.1016/S0006-3495(94)80500-4 7919018 PMC1225377

[37] Murphy RM, Larkins NT, Mollica JP, Beard NA, Lamb GD. Calsequestrin content and SERCA determine normal and maximal Ca ^2+^ storage levels in sarcoplasmic reticulum of fast- and slow-twitch fibres of rat: Calsequestrin and SR Ca ^2+^ content in single muscle fibres. J Physiol. 2009;587: 443–460. 10.1113/jphysiol.2008.163162 19029185 PMC2670055

[38] Fénelon K, Lamboley CRH, Carrier N, Pape PC. Calcium buffering properties of sarcoplasmic reticulum and calcium-induced Ca2+ release during the quasi-steady level of release in twitch fibers from frog skeletal muscle. J Gen Physiol. 2012;140: 403–419. 10.1085/jgp.201110730 23008434 PMC3457687

[39] Weiss JN. The Hill equation revisited: uses and misuses. FASEB J Off Publ Fed Am Soc Exp Biol. 1997;11: 835–841. 9285481

[40] Park H, Park IY, Kim E, Youn B, Fields K, Dunker AK, et al Comparing skeletal and cardiac calsequestrin structures and their calcium binding: a proposed mechanism for coupled calcium binding and protein polymerization. J Biol Chem. 2004;279: 18026–18033. 10.1074/jbc.M311553200 14871888

[41] Fliegel L, Burns K, MacLennan DH, Reithmeier RA, Michalak M. Molecular cloning of the high affinity calcium-binding protein (calreticulin) of skeletal muscle sarcoplasmic reticulum. J Biol Chem. 1989;264: 21522–21528. 2600080

[42] Ostwald TJ, MacLennan DH. Isolation of a high affinity calcium-binding protein from sarcoplasmic reticulum. J Biol Chem. 1974;249: 974–979. 4272851

[43] Palmer AE, Jin C, Reed JC, Tsien RY. Bcl-2-mediated alterations in endoplasmic reticulum Ca2+ analyzed with an improved genetically encoded fluorescent sensor. Proc Natl Acad Sci. 2004;101: 17404–17409. 10.1073/pnas.0408030101 15585581 PMC535104

[44] Selvin D, Hesse E, Renaud J-M. Properties of single FDB fibers following a collagenase digestion for studying contractility, fatigue, and pCa-sarcomere shortening relationship. Am J Physiol—Regul Integr Comp Physiol. 2015;308: R467–R479. 10.1152/ajpregu.00144.2014 25568074 PMC4360063

[45] Michelucci A, Paolini C, Boncompagni S, Canato M, Reggiani C, Protasi F. Strenuous exercise triggers a life-threatening response in mice susceptible to malignant hyperthermia. FASEB J. 2017; fj.201601292R. 10.1096/fj.201601292R 28465322 PMC5503704

[46] Michalak M, Corbett EF, Mesaeli N, Nakamura K, Opas M. Calreticulin: one protein, one gene, many functions. Biochem J. 1999;344 Pt 2: 281–292. 10567207 PMC1220642

[47] Rossi AE, Dirksen RT. Sarcoplasmic reticulum: the dynamic calcium governor of muscle. Muscle Nerve. 2006;33: 715–731. 10.1002/mus.20512 16477617

[48] Yoshida M, Minamisawa S, Shimura M, Komazaki S, Kume H, Zhang M, et al Impaired Ca2+ store functions in skeletal and cardiac muscle cells from sarcalumenin-deficient mice. J Biol Chem. 2005;280: 3500–3506. 10.1074/jbc.M406618200 15569689

[49] Divet A, Paesante S, Grasso C, Cavagna D, Tiveron C, Paolini C, et al Increased Ca2+ storage capacity of the skeletal muscle sarcoplasmic reticulum of transgenic mice over-expressing membrane bound calcium binding protein junctate. J Cell Physiol. 2007;213: 464–474. 10.1002/jcp.21121 17516551

[50] Lamboley CRH, Kake Guena SA, Touré F, Hébert C, Yaddaden L, Nadeau S, et al New method for determining total calcium content in tissue applied to skeletal muscle with and without calsequestrin. J Gen Physiol. 2015;145: 127–153. 10.1085/jgp.201411250 25624449 PMC4306712

[51] Y V et al Accelerated activation of SOCE current in myotubes from two mouse models of anesthetic- and heat-induced sudden death.—PubMed—NCBI [Internet]. [cited 15 Nov 2017]. https://www.ncbi.nlm.nih.gov/pubmed/24143248 10.1371/journal.pone.0077633 PMC3797063 24143248

[52] Boncompagni S, Michelucci A, Pietrangelo L, Dirksen RT, Protasi F. Exercise-dependent formation of new junctions that promote STIM1-Orai1 assembly in skeletal muscle. Sci Rep. 2017;7: 14286 10.1038/s41598-017-14134-0 29079778 PMC5660245

[53] Launikonis BS, Ríos E. Store-operated Ca2+ entry during intracellular Ca2+ release in mammalian skeletal muscle. J Physiol. 2007;583: 81–97. 10.1113/jphysiol.2007.135046 17569733 PMC2277221

[54] Vecellio Reane D, Vallese F, Checchetto V, Acquasaliente L, Butera G, De Filippis V, et al A MICU1 Splice Variant Confers High Sensitivity to the Mitochondrial Ca2+ Uptake Machinery of Skeletal Muscle. Mol Cell. 2016;64: 760–773. 10.1016/j.molcel.2016.10.001 27818145

[55] Cortassa S, Aon MA, Marbán E, Winslow RL, O’Rourke B. An Integrated Model of Cardiac Mitochondrial Energy Metabolism and Calcium Dynamics. Biophys J. 2003;84: 2734–2755. 10.1016/S0006-3495(03)75079-6 12668482 PMC1201507

[56] Tran K, Smith NP, Loiselle DS, Crampin EJ. A Thermodynamic Model of the Cardiac Sarcoplasmic/Endoplasmic Ca2+ (SERCA) Pump. Biophys J. 2009;96: 2029–2042. 10.1016/j.bpj.2008.11.045 19254563 PMC2717298

