# Supplementary material for: A 3D diffusional-compartmental model of the calcium dynamics in cytosol, sarcoplasmic reticulum and mitochondria of murine skeletal muscle fibers
Source: PLoS One. 2018 Jul 26;13(7):e0201050. doi: 10.1371/journal.pone.0201050 (PMC6062086; doi:10.1371/journal.pone.0201050)
Supplement: S1 Table — (PDF) [file pone.0201050.s001.pdf]

Table S1: Parameters definitions and values.

Parameter	Meaning	Value	Reference
L	Length of half-sarcomere	1.25 μm	
R	Radius of sarcomere	0.5 μm	
V_{tot}	Half-sarcomere volume	π R^2 L	-
V_{TC}	Volume of TC	3.5% V_{tot}	(1)
V_{SR}	Volume of SR	5.5% V_{tot}	(1)
V_{MF}	Myofibrillar volume	80% V_{tot}	(1)
V_{mito}	Mitochondrion volume	5% V_{tot}	(1)
V_{EF}	Extra-myofibrillar volume	6% V_{tot}	(1)
D	Free diffusion coefficient	300 μm^2 s^{-1}	(2)
[CSQ]	Calsequestrin concentration	82.8 mM	(3)
k^{CS}_{ON}	Calsequestrin binding rate	3 10^{-3} μM^{-1} s^{-1}	(4)
k^{CS}_{OFF}	Calsequestrin unbinding rate	3 s^{-1}	(4)
h^{CSQ}	Calsequestrin Hill coefficient	3	(5)
[AB]	Additional buffer concentration	10% or 20% of [CSQ]	(5)
K^{AB}_{ON}	Additional buffer binding rate	1.5 10^{-3} μM^{-1} s^{-1}	This study
K^{AB}_{OFF}	Additional buffer unbinding rate	2.25 10^{-6} s^{-1}	This study
PVA	Parvalbumin concentration	1000 μM	(4)
k^{PVA}_{ON}	Parvalbumin binding rate	4.5 10^{2} μM^{-1} s^{-1}	(4)
k^{PVA}_{OFF}	Parvalbumin unbinding rate	1 s^{-1}	(4)
Tn	Troponin concentration	240 μM	(2)
k^{Tr}_{ON}	Troponin binding rate	0.885 10^{2} μM^{-1} s^{-1}	(2)
k^{Tr}_{OFF}	Troponin unbinding rate	115 s^{-1}	(2)
Mg^{2+}	Mg^{2+} concentration	3300 μM	(4)
k^{Mg}_{ON}	Mg^{2+} binding rate to Parvalbumin	1.2 10^{-1} μM^{-1} s^{-1}	(4)
k^{Mg}_{OFF}	Mg^{2+} unbinding rate to Parvalbumin	3.4 s^{-1}	(4)
B	Mitochondrion buffer	2 (or 20) μM/V_{tot}	(6)
k^{B}_{ON}	Mitochondrion buffer binding rate	0.8 μM^{-1} s^{-1}	This study
k^{B}_{OFF}	Mitochondrion buffer unbinding rate	0.192 s^{-1}	As (7)
T	Absolute temperature	298 K	As (8)
f_{NCE}	Multiplication factor NCE	0.92	This study
$\Delta\Psi_m$	Mitochondrial inner potential	190 mV	(6)
K^{NCE}_{Ca}	Ca^{2+} binding constant of NCE	1.1 μM	This study
f_{MCU}	Multiplication factor MCU	10^2	This study
V_{MCU}	Maximum flux rate MCU	2 μM s^{-1}	(6)
h	MCU Hill coefficient	2	This study
K_d	MCU half-maximum pump [Ca^{2+}]	1 μM	This study
V_{max}	Maximum pump rate for SERCA	4 10^3 μM/V_{tot} s^{-1}	(9)
K_m	SERCA half-maximum [Ca^{2+}]	0.5 μM	(9)
P_{max}	Maximum RyR "evacuability"	575 s^{-1}	This study
τ_{ON}	Rise time constant in RyR permeability	1 ms^{-1}	(1)
τ_{OFF}	Decay time constant in RyR permeability	5 ms^{-1}	(1)

References:

1. Cannell, M.B., and D.G. Allen. 1984. Model of calcium movements during activation in the sarcomere of frog skeletal muscle. Biophys. J. 45: 913–925.

2. Baylor, S.M., and S. Hollingworth. 2007. Simulation of Ca^{2+} Movements within the Sarcomere of Fast-Twitch Mouse Fibers Stimulated by Action Potentials. J. Gen. Physiol. 130: 283–302.

3. Murphy, R.M., N.T. Larkins, J.P. Mollica, N.A. Beard, and G.D. Lamb. 2009. Calsequestrin content and SERCA determine normal and maximal Ca^{2+} storage levels in sarcoplasmic reticulum of fast- and slow-twitch fibres of rat: Calsequestrin and SR Ca^{2+} content in single muscle fibres. J. Physiol. 587: 443–460.

4. Westerblad, H., and D.G. Allen. 1994. The role of sarcoplasmic reticulum in relaxation of mouse muscle; effects of 2, 5-di (tert-butyl)-1, 4-benzohydroquinone. J. Physiol. 474: 291.

5. Fénelon, K., C.R.H. Lamboley, N. Carrier, and P.C. Pape. 2012. Calcium buffering properties of sarcoplasmic reticulum and calcium-induced Ca2+ release during the quasi-steady level of release in twitch fibers from frog skeletal muscle. J. Gen. Physiol. 140: 403–419.

6. Wüst, R.C.I., M. Helmes, J.L. Martin, T.J.T. van der Wardt, R.J.P. Musters, J. van der Velden, and G.J.M. Stienen. 2017. Rapid frequency-dependent changes in free mitochondrial calcium concentration in rat cardiac myocytes: Mitochondrial calcium handling. J. Physiol. 595: 2001–2019.

7. Scorzeto, M., M. Giacomello, L. Toniolo, M. Canato, B. Blaauw, C. Paolini, F. Protasi, C. Reggiani, and G.J.M. Stienen. 2013. Mitochondrial Ca2+-Handling in Fast Skeletal Muscle Fibers from Wild Type and Calsequestrin-Null Mice. PLoS ONE. 8: e74919.

8. Canato, M., M. Scorzeto, M. Giacomello, F. Protasi, C. Reggiani, and G.J.M. Stienen. 2010. Massive alterations of sarcoplasmic reticulum free calcium in skeletal muscle fibers lacking calsequestrin revealed by a genetically encoded probe. Proc. Natl. Acad. Sci. 107: 22326–22331.

9. Lytton, J., M. Westlin, S.E. Burk, G.E. Shull, and D.H. MacLennan. 1992. Functional comparisons between isoforms of the sarcoplasmic or endoplasmic reticulum family of calcium pumps. J. Biol. Chem. 267: 14483–14489.
