# Supplementary material for: A 3D diffusional-compartmental model of the calcium dynamics in cytosol, sarcoplasmic reticulum and mitochondria of murine skeletal muscle fibers
Source: PLoS One. 2018 Jul 26;13(7):e0201050. doi: 10.1371/journal.pone.0201050 (PMC6062086; doi:10.1371/journal.pone.0201050)
Supplement: S2 Table — To evaluate the sensitivity of the model predictions on the actual model parameters, a change of ±5% has been applied to each parameter reported in Table 2, relative to the value given in Table 1. The variations of free [Ca2+] in the three compartments (cytosol, SR, mitochondrion) using these modified parameters ([Ca2+]±5%) relative to the values obtained with the parameter values in Table 1 ([Ca2+]basic) are calculated and expressed as a percentage (-5%/+5%). All percentages are lower than 5% except for the Hill parameter (an exponential term) in the calsequestrin equilibrium equation. The values of 100∙([Ca2+]5%[Ca2+]basic-1) in the three compartments are reported. “-”means less than 0.1% deviation. Values are given for the -5%/+5% cases. (PDF) [file pone.0201050.s002.pdf]

S2 Table. Sensitivity analysis. To evaluate the sensitivity of the model predictions on the actual model parameters, a change of ±5% has been applied to each parameter reported in Table 2, relative to the value given in Table 1. The variations of free [Ca^{2+}] in the three compartments (cytosol, SR, mitochondrion) using these modified parameters (*[Ca^{2+}]$_{\pm5\%}$*) relative to the values obtained with the parameter values in Table 1 (*[Ca^{2+}]$_{basic}$*) are calculated and expressed as a percentage (-%5/+5%). All percentages are lower than 5% except for the Hill parameter (an exponential term) in the calsequestrin equilibrium equation. The values of $100 \cdot (\frac{[Ca^{2+}]_{5\%}}{[Ca^{2+}]_{basic}} - 1)$ in the three compartments are reported.
"-" means less than 0.1% deviation. Values are given for the -5%/+5% cases

Parameter	Cytosol	SR	Mitochondrion
k^{CS}_{ON}	1.2/-1.2	0.9/-0.8	0.1/-0.2
k^{CS}_{OFF}	-1.2/1.2	-0.9/0.8	-0.2/0.1
h^{CSQ}	-35.2/-19.6	-31.4/-15.0	-6.6/-3.1
AB	0.2/-0.3	0.2/-0.2	-/-
K^{AB}_{ON}	-/-	-/-	-/-
K^{AB}_{OFF}	-0.2/0.2	-0.1/0.2	-/-
PVA	4.3/-4.1	3.0/-2.9	0.6/-0.6
k^{PVA}_{ON}	1.8/-1.7	1.3/-1.2	0.2/-0.3
k^{PVA}_{OFF}	-1.7/1.6	-1.2/1.1	-0.2/0.2
Tn	0.4/-0.4	0.2/-0.2	-/-0.1
k^{Tr}_{ON}	0.3/-0.3	0.2/-0.2	-/-0.1
k^{Tr}_{OFF}	-0.2/0.2	-0.2/0.2	-/-
Mg^{2+}	-1.8/1.7	-1.3/1.2	-0.2/0.2
k^{Mg}_{ON}	-1.8/1.7	-1.3/1.2	-0.3/0.2
k^{Mg}_{OFF}	1.9/-1.7	1.4/-1.3	0.2/-0.3
B	-/0.1	-/0.4	-/-
k^{B}_{ON}	-/-	-/-	-/-
k^{B}_{OFF}	-/-	-/-	-/-
f_{NCE}	-/-	-/-	0.4/-0.4
$\Delta\Psi_m$	-/-	-/-	1.4/-1.4
K^{NCE}_{Ca}	-/-	-/-	-0.2/0.2
$[Ca^{2+}]_{th}$	-/-	-/-	-4.0/4.0
γ	-/-	-/-	0.8/-0.7
V_{MCU}	-/-	-/-	-0.4/0.3
h	-/-	-/-	0.3/-0.4
K_d	-/-	-/-	0.5/-0.5
V_{max}	4.1/-3.5	-1.5/2.2	0.6/-0.5
K_m	-0.9/1.1	0.4/-	-0.1/0.2
P_{max}	-4.0/4.1	2.0/-2.0	-0.6/0.5
