# Supplementary material for: A 3D diffusional-compartmental model of the calcium dynamics in cytosol, sarcoplasmic reticulum and mitochondria of murine skeletal muscle fibers
Source: PLoS One. 2018 Jul 26;13(7):e0201050. doi: 10.1371/journal.pone.0201050 (PMC6062086; doi:10.1371/journal.pone.0201050)
Supplement: S1 Fig — Simulation of the [Ca2+] in the cytosol where NCE and MCU are supposed to be located (125 nm away from the RyR) and in the mitochondrion using the 50-compartments model (red) and a 300-compartments model (blue). (PDF) [file pone.0201050.s003.pdf]

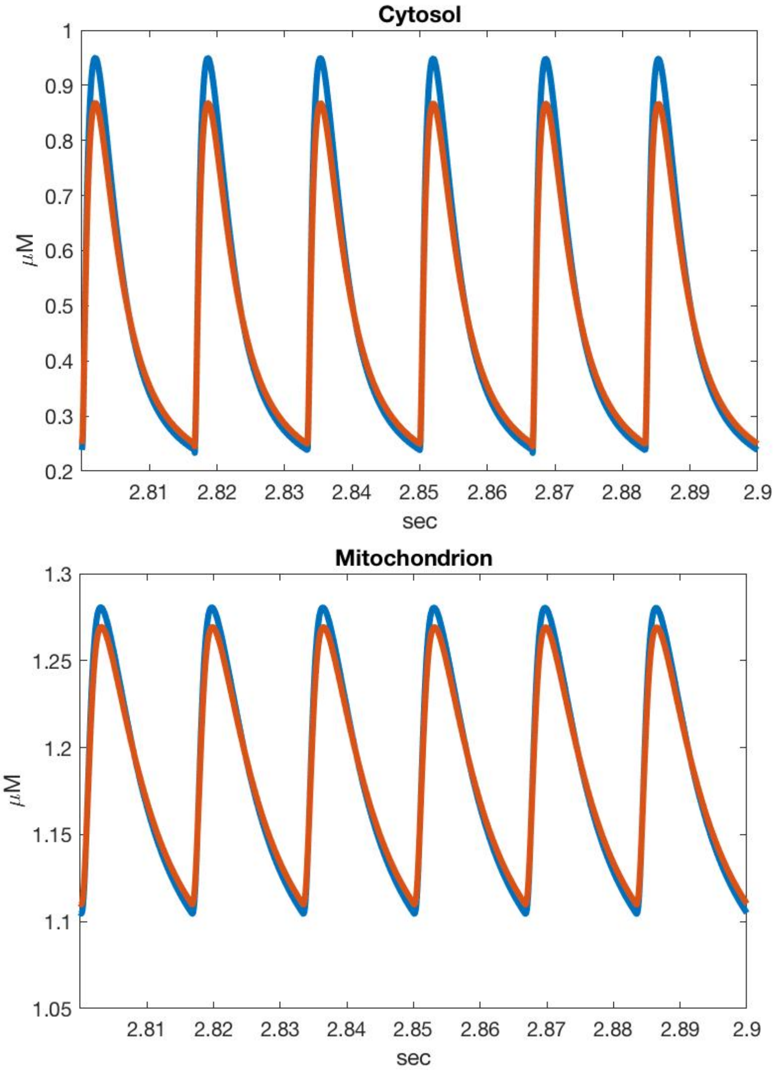

S1 Fig. Effect of the compartment dimension. Simulation of the [Ca2+] in the cytosol where NCE and MCU are supposed to be located (125 nm away from the RyR) and in the mitochondrion using the 50-compartments model (red) and a 300-compartments model (blue).
