# Supplementary material for: A 3D diffusional-compartmental model of the calcium dynamics in cytosol, sarcoplasmic reticulum and mitochondria of murine skeletal muscle fibers
Source: PLoS One. 2018 Jul 26;13(7):e0201050. doi: 10.1371/journal.pone.0201050 (PMC6062086; doi:10.1371/journal.pone.0201050)
Supplement: S1 File — Effect of the Ca2+ dependent inactivation on the model prediction. (PDF) [file pone.0201050.s004.pdf]

S1 File

Ca^{2+}-dependent inactivation of SR Ca^{2+}-release

A negative feedback mechanisms, called Ca^{2+} dependent inactivation, has been shown in mouse, and frog skeletal muscle (1). It implies that the release flux is strongly reduced in the second and subsequent action potentials (AP) compared to the first one if the frequency is high enough and thus the interval between action potentials is enough short. The flux has been estimated to decline to 25% passing from the first AP to the second, and to 15% of the initial value during the fifth AP (1). In the present model the value of P_{max} is constant and does not depend on $[Ca^{2+}]_{TC}$ nor on $[Ca^{2+}]_{cyto}$. The model parameters, P_{max} included, have been defined to fit the steady state values of calcium concentrations in the three compartments. Fig S9 illustrates the difference in the $[Ca^{2+}]_{cyto}$ transients with Ca^{2+}-dependent inactivation as described in (1) and without Ca^{2+}-dependent inactivation, i.e. with P_{max} constant. They differ only for the first APs, and almost superimpose after the fifth.

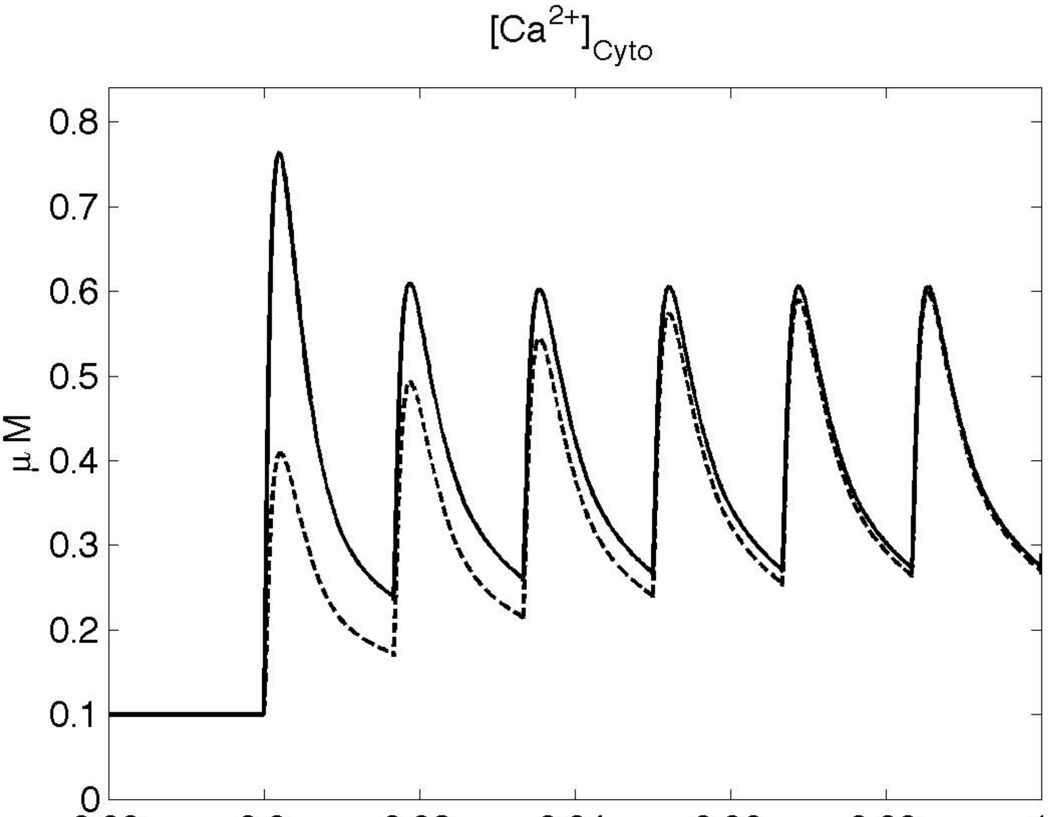

S2 Fig. Ca^{2+}-dependent inactivation of SR Ca^{2+}-release. Sequence of the first six $[Ca^{2+}]_{cyto}$ transients in the model (dashed line) is obtained without considering the Ca^{2+}-dependent inactivation. It differs from the trace simulated in a model including this effect (continuous line) only for the first few action potentials.

References:

1. Baylor, S.M., and S. Hollingworth. 2007. Simulation of Ca $^{2+}$ Movements within the Sarcomere of Fast-Twitch Mouse Fibers Stimulated by Action Potentials. J. Gen. Physiol. 130: 283–302.
