# Supplementary material for: A 3D diffusional-compartmental model of the calcium dynamics in cytosol, sarcoplasmic reticulum and mitochondria of murine skeletal muscle fibers
Source: PLoS One. 2018 Jul 26;13(7):e0201050. doi: 10.1371/journal.pone.0201050 (PMC6062086; doi:10.1371/journal.pone.0201050)
Supplement: S2 File — Equations for the MCU and NCE fluxes in mitochondrion and phenomenological hypothesis. (PDF) [file pone.0201050.s005.pdf]

S2 File.

Mitochondrion modeling.

Ca^{2+}-handling by the mitochondria is described starting from the model proposed in (1) for cardiac myocytes. In the model $[Ca^{2+}]_{mito}$ is regulated by an influx through the MCU, an efflux through the NCE and internal buffering. The efflux has been defined according to the 3:1 stoichiometry for Na^+ and Ca^{2+} proposed in (2):

$$J_{NCE} = f_{NCE} V_{NCE} \left(\frac{e^{+\frac{0.5\Delta\Psi_m F}{RT}} \frac{[Na^+]_x^3 [Ca^{2+}]_m}{K_{Na}^3 K_{Ca}} - e^{-\frac{0.5\Delta\Psi_m F}{RT}} \frac{[Na^+]_m^3 [Ca^{2+}]_x}{K_{Na}^3 K_{Ca}}}{1 + \frac{[Na^+]_x^3}{K_{Na}^3} + \frac{[Ca^{2+}]_m}{K_{Ca}} + \frac{[Na^+]_x^3 [Ca^{2+}]_m}{K_{Na}^3 K_{Ca}} + \frac{[Na^+]_m^3}{K_{Na}^3} + \frac{[Ca^{2+}]_x}{K_{Ca}} + \frac{[Na^+]_m^3 [Ca^{2+}]_x}{K_{Na}^3 K_{Ca}}} \right)$$

(S1)

where the subscript x stands for the sub-compartment of EF one element apart from TC (125 nm away from RyR), and the subscript m stands for the inner mitochondrial space. V_{NCE} is the activity for NCE and f_{NCE} is a multiplication factor. Influx of Ca^{2+} ions within the mitochondrion is described in Eq. 5 in the main text, through a simplified description of MCU as a saturable first-order transport mechanism independent of the internal $[Ca^{2+}]_{mito}$ and constant mitochondrial membrane potential. This choice limits the matching of the fast rise in $[Ca^{2+}]_{mito}$ during stimuli with the slower decay after stimuli (see (3)). To explain this limitation, we can start writing the governing equation for $[Ca^{2+}]_{mito}$ as:

$$\frac{\partial [Ca^{2+}]_{mito}}{\partial t} = J_{MCU}([Ca^{2+}]_{cyto}) - J_{NCE}([Ca^{2+}]_{mito}, [Ca^{2+}]_{cyto})$$

(S2)

Cytosolic calcium kinetics are considerably faster than the calcium kinetics in the mitochondrion, both during and after the stimulation trains. Therefore, to simplify the description, we can consider the behavior of the $[Ca^{2+}]_{mito}$ against a square pulse wave change in cytosolic concentration. J_{MCU} assumes two constant values during and after the stimuli, defined as C_R and C_S respectively. Assuming a linear dependence of J_{NCE} on $[Ca^{2+}]_{mito}$, $J_{NCE}=k_{out} [Ca^{2+}]_{mito}$, the analytical solution can be written as:

$$[Ca^{2+}]_{mito}(t) = \frac{C_S}{k_{out}} [Ca^{2+}]_{cyto} (1 - e^{-k_{out} t})$$

(S3)

in the rising phase ($[Ca^{2+}]_{cyto} > [Ca^{2+}]_{mito} \approx 0$) till the decay time t_{decay}, and as:

$$[Ca^{2+}]_{mito}(t - t_{decay}) = [Ca^{2+}]_{mito}(t_{decay})(e^{-k_{out}(t - t_{decay})})$$

(S4)

in the decay phase ($[Ca^{2+}]_{mito} > [Ca^{2+}]_{cyto} \approx 0$). Therefore, in this case the kinetics of the two phases are identical. A more complicated situation occurs when we impose J_{NCE} as described above. However, the values imposed for the mitochondria membrane potential, make the

second term in the nominator almost negligible. Moreover, in the simplified square pulse case, all the terms with $[Ca^{2+}]_{cyto}$ in the denominator are constant and J_{NCE} becomes a less than linear function of $[Ca^{2+}]_{mito}$ which is not compatible with different rate constants for the rising and decay phase (Fig S1, green line). However, if we introduce a modified version of J_{NCE}, by multiplying it by a factor S_{NCE}:

$$S_{NCE} = \begin{cases} 1, & if \quad [Ca^{2+}]_{mito} < [Ca^{2+}]_{th} \\ \exp(\gamma[Ca^{2+}]_{mito})/C, & if \quad [Ca^{2+}]_{mito} \geq [Ca^{2+}]_{th} \end{cases}$$

(S5)

In this case, J_{NCE} has an approximately linear increase up to the threshold value $[Ca^{2+}]_{th}$= 1 μM, and increases exponentially above this value. The parameter *C* and a value of γ as given in Table S2 makes J_{NCE} continuous (see Fig S1) and results in different kinetics during the rising and decay phase corresponding to the experimental findings as described in the main text .

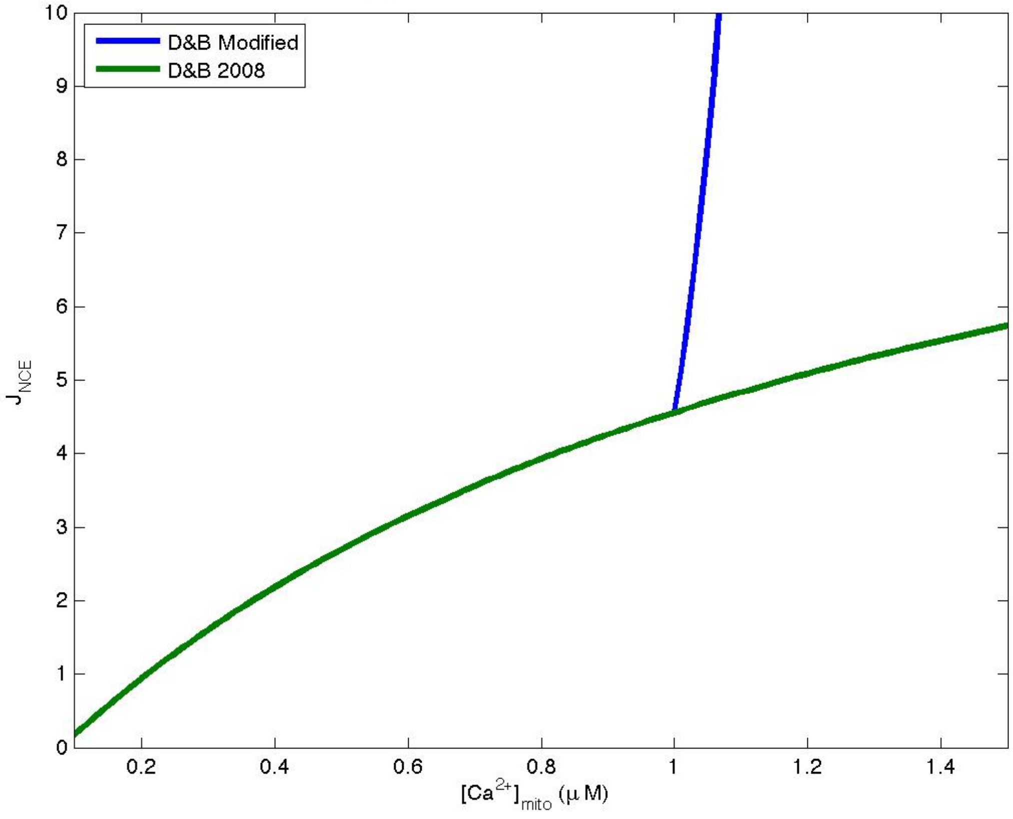

Fig S3: The original and the modified NCE flux derived from the Dash & Beard 2008 model. The modified flux ensures a proper amount of $[Ca^{2+}]_{mito}$ and a slow return of the signal after train of stimuli. An alternative modification that would exert the same effect would be to slow MCU influx at a certain, probably similar, $[Ca^{2+}]_{mito}$ threshold value.

References

1. Wüst, R.C.I., M. Helmes, J.L. Martin, T.J.T. van der Wardt, R.J.P. Musters, J. van der Velden, and G.J.M. Stienen. 2017. Rapid frequency-dependent changes in free mitochondrial calcium concentration in rat cardiac myocytes: Mitochondrial calcium handling. J. Physiol. 595: 2001–2019.

2. Dash, R.K., and D.A. Beard. 2008. Analysis of cardiac mitochondrial Na^{+}-Ca^{2+} exchanger kinetics with a biophysical model of mitochondrial Ca^{2+} handing suggests a 3: 1 stoichiometry: Characterization of mitochondrial NCE stoichiometry. J. Physiol. 586: 3267–3285.

3. Scorzeto, M., M. Giacomello, L. Toniolo, M. Canato, B. Blaauw, C. Paolini, F. Protasi, C. Reggiani, and G.J.M. Stienen. 2013. Mitochondrial Ca2+-Handling in Fast Skeletal Muscle Fibers from Wild Type and Calsequestrin-Null Mice. PLoS ONE. 8: e74919.
