# Supplementary material for: A 3D diffusional-compartmental model of the calcium dynamics in cytosol, sarcoplasmic reticulum and mitochondria of murine skeletal muscle fibers
Source: PLoS One. 2018 Jul 26;13(7):e0201050. doi: 10.1371/journal.pone.0201050 (PMC6062086; doi:10.1371/journal.pone.0201050)
Supplement: S3 File — Although the model was designed mainly to simulate the steady state concentrations reached at rest or during stimulation trains in the three main compartments, also the kinetics after the train of stimuli were well simulated, both in SR and in mitochondrion. (PDF) [file pone.0201050.s006.pdf]

S3 File. Analysis of the kinetics of the decay phase of the transients in sarcoplasmic reticulum and mitochondrion. Although the model was designed mainly to simulate the steady state concentrations reached at rest or during stimulation trains in the three main compartments, also the kinetics after the train of stimuli were well simulated, both in SR (S4 Fig.) and in mitochondrion (S5 Fig.).

$[Ca^{2+}]_{mito}$ shows an initial phase of fast decay before reaching the single exponential behavior in the decay after the train of stimuli. It is related to the uptake of Ca^{2+} ions from the buffer as shown in S5 Fig.. A similar behavior has been seen also experimentally as shown in (1).

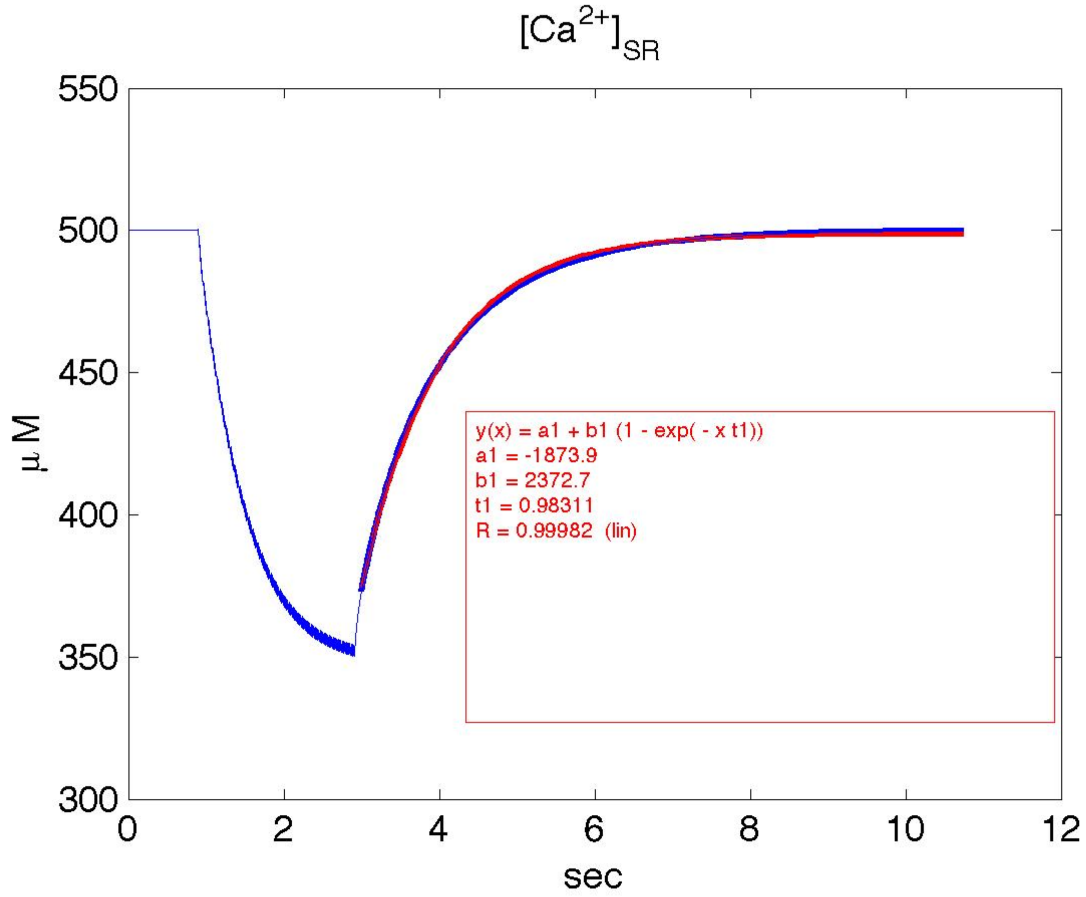

S4 Fig. Kinetics of $[Ca^{2+}]$ re-uptake. The kinetics of the $[Ca^{2+}]$ re-uptake in the SR is fitted by a single exponential with time constant t1=0.98 s^{-1}. This value is higher, but not very far from the experimental value of 0.3 s^{-1}, see (2).

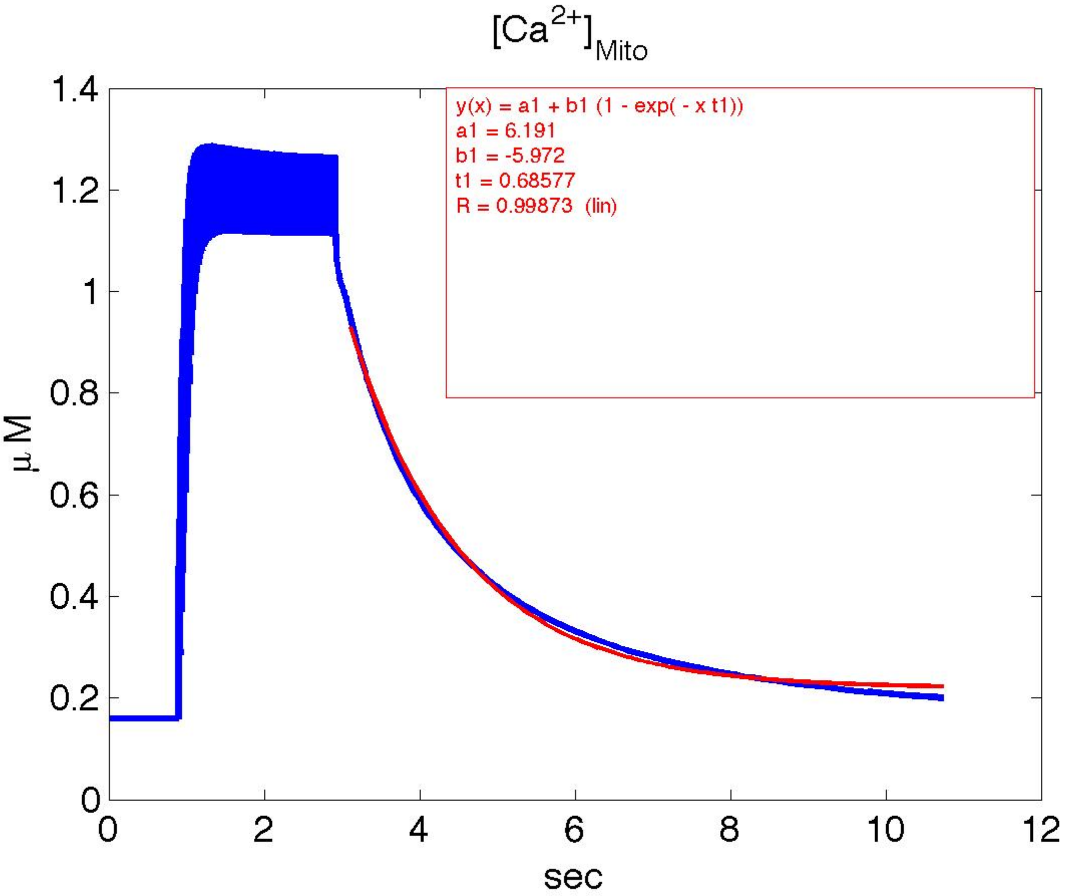

S5 Fig. $[Ca^{2+}]_{mito}$ decay. The slower phase of decay in the $[Ca^{2+}]_{mito}$ is governed by a single exponential with time constant of 0.69 s^{-1} which is not far from the experimental value of 0.21 s^{-1} (see (1))

References:

1. Scorzeto, M., M. Giacomello, L. Toniolo, M. Canato, B. Blaauw, C. Paolini, F. Protasi, C. Reggiani, and G.J.M. Stienen. 2013. Mitochondrial Ca2+-Handling in Fast Skeletal Muscle Fibers from Wild Type and Calsequestrin-Null Mice. PLoS ONE. 8: e74919.

2. Canato, M., M. Scorzeto, M. Giacomello, F. Protasi, C. Reggiani, and G.J.M. Stienen. 2010. Massive alterations of sarcoplasmic reticulum free calcium in skeletal muscle fibers lacking calsequestrin revealed by a genetically encoded probe. Proc. Natl. Acad. Sci. 107: 22326–22331.
