# Supplementary material for: A 3D diffusional-compartmental model of the calcium dynamics in cytosol, sarcoplasmic reticulum and mitochondria of murine skeletal muscle fibers
Source: PLoS One. 2018 Jul 26;13(7):e0201050. doi: 10.1371/journal.pone.0201050 (PMC6062086; doi:10.1371/journal.pone.0201050)
Supplement: S4 File — The nature of the mitochondrial buffer is largely unknown at present, and experimental data needed for its mathematical characterization are not available in literature. We have analyzed the [Ca2+]mito behavior for different choices of the buffering capacity and kinetics, keeping a ratio for free-to-bound [Ca2+] of 1:100. (PDF) [file pone.0201050.s007.pdf]

S4 File. Sensitivity analysis of the mitochondrial buffer parameters. The nature of the mitochondrial buffer is largely unknown at present, and experimental data needed for its mathematical characterization are not available in literature. We have analyzed the $[Ca^{2+}]_{mito}$ behavior for different choices of the buffering capacity and kinetics, keeping a ratio for free-to-bound $[Ca^{2+}]$ of 1:100 (1).

Varying the kinetics of the binding-unbinding of Ca^{2+} to the buffer, does not introduce a large difference in the overall behavior, as shown in Fig S6 where both the binding and unbinding rate constants have been multiplied by a factor of 0.3 and 3, respectively. Small variations are mainly observed during the rising phase and during the decay after the end of the train of stimuli.

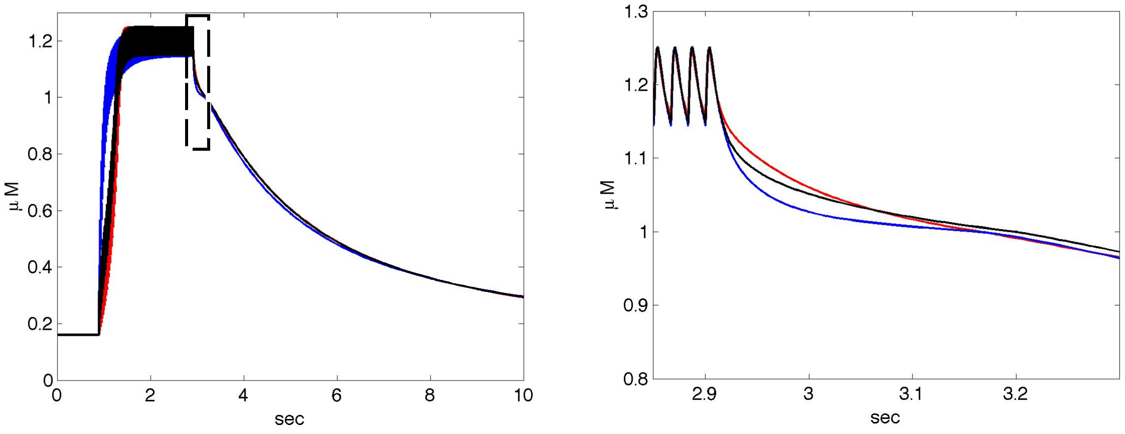

S6 Fig.: Effect of the inner mitochondrial buffer kinetics on the $[Ca^{2+}]_{mito}$. The predictions with the parameter values (see Table S1) used in the model described in the text (black line) are shown together with the predictions with three-fold faster (red line) and three-fold slower (blue line) kinetics for mitochondrial buffer binding and unbinding of Ca^{2+}. Steady state values reached during a 60 Hz, 2 seconds stimulation train are not much affected, as well as the slow decay after the end of the train as it depends mainly on J_{NCE}. On the contrary, there is a clear impact on the rise phase and the initial fast part in the decay (the window in the left panel is enlarged in the right panel).

We have also tested larger buffer concentration, increasing [B] from 2 to 20 μM, while keeping the rate constants fixed. As can be seen in Fig S7, the kinetics in the rising and decay phases of $[Ca^{2+}]_{mito}$ transient are affected by the concentration of the mitochondrial buffer, as well as the predicted oscillations, while its average amplitude is maintained approximatively constant because of our choice of J_{MCU} and J_{NCE}.

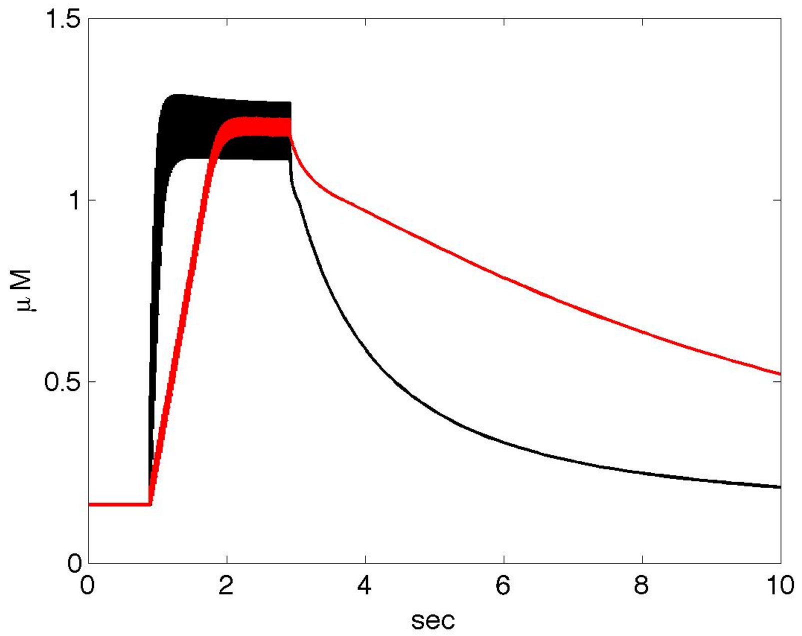

S7 Fig. Effect of the inner mitochondrial buffer quantity. Free $[Ca^{2+}]_{mito}$ traces for two different values of [B], 2 µM (black line) and 20 µM (red line). The average value is maintained constant regardless the total buffer concentration.

References:

1. Wüst, R.C.I., M. Helmes, J.L. Martin, T.J.T. van der Wardt, R.J.P. Musters, J. van der Velden, and G.J.M. Stienen. 2017. Rapid frequency-dependent changes in free mitochondrial calcium concentration in rat cardiac myocytes: Mitochondrial calcium handling. J. Physiol. 595: 2001–2019.
