# Supplementary material for: A 3D diffusional-compartmental model of the calcium dynamics in cytosol, sarcoplasmic reticulum and mitochondria of murine skeletal muscle fibers
Source: PLoS One. 2018 Jul 26;13(7):e0201050. doi: 10.1371/journal.pone.0201050 (PMC6062086; doi:10.1371/journal.pone.0201050)
Supplement: S5 File — Effect of the kinetic term on the [Ca2+] in the three compartments. (PDF) [file pone.0201050.s008.pdf]

Supporting Information S9

Kinetics and steady state determination of Ca^{2+} concentrations

Ca^{2+} concentrations considered in the paper have been derived from previously published studies from the fluorescence ratio (F) of Fura-2 in cytosolic space, or the Förster resonance energy transfer (FRET) intensity ratio (R=YFP/CFP) of genetically targeted indicators (Cameleon) directed either to the SR (D1ER) or to the mitochondria (4mtD3cpv). Data collection methods are reported in [1] and [2], respectively.
The equation used to determine the [Ca^{2+}] transient from cytosolic fluorescence ratio F proposed by Klein and colleagues [3] is:

$$[Ca^{2+}](t) = \frac{dF/_{dt} + k_{off}(F - F_{min})}{k_{on}(F_{max} - F)}$$

(S6)

in which F_{min} and F_{max} indicate the minimum (calcium depleted) and maximum (calcium saturated) value of F.
As to the Cameleon ratio R, we extend the derivation proposed by Sztretye et al. [4] in the case of n=1. In our case calibration curves for mitochondrial and reticular cameleon (Table S3) indicate cooperative binding of Ca^{2+} with the probe S with n≠1 and:

$$\frac{d[nCa^{2+}S]}{dt} = k_{on}[Ca^{2+}]^n[S] - k_{off}[nCa^{2+}S]$$

(S7)

For dimensional reasons, k_{on} in this equation must be expressed in $M^{-n}s^{-1}$. The corresponding equation for [Ca^{2+}](t) becomes:

$$[Ca^{2+}](t) = K'_d\left[\frac{1}{k_{off}}\frac{dR}{dt}\frac{R_{max} - R_{min}}{[(R_{max} - R) + \beta(R - R_{min})](R_{max} - R)} + \frac{R - R_{min}}{R_{max} - R}\right]^{1/n}$$

(S8)

in which R_{min} and R_{max} indicate the minimum (calcium-free) and maximum (calcium-saturated) value of R and β is the ratio of the Ca^{2+}-free/Ca^{2+}-saturated CFP fluorescence. According to Palmer et al [5] K'_d in equation S8 is the apparent equilibrium constant expressed in M^{-1} which includes the β term. In this case, analogous to the equations proposed by Klein et al [3} and Sztretye et al. [4], the first (kinetic) term between the brackets containing dR/dt represents the difference with the quasi steady-state term.
An example of the free calcium concentrations calculated from the Fura-2 signals during a 2 s tetanus at 60 Hz with the complete equation including the kinetic term and a simplified equation with only the steady state component is reported in S8 Fig. As can be seen the influence of the kinetic term is small.
Unfortunately, no detailed information is available on the calcium on-off kinetics of the cameleon probes D1ER and 4mtD3cpv. However, using for D1ER a value for k_{off}=256 s^{-1} proposed in [5], the influence of the kinetic term is almost negligible (S9 Fig.). Only unrealistic values of three orders of magnitude lower would yield a noticeable difference between the two curves.

To the best of our knowledge, the k_{off} of 4mtD3cpv has not been determined yet. However, a very small value of k_{off} must be used to observe a small discrepancy due to the kinetic term relative to the quasi-steady state term. An example, assuming k_{off}=7.6 s^{-1} is shown S10 Fig.
It is, moreover, important to underline that, in this study, we have compared the simulated concentrations to the experimental data observed in the steady-state during a train of stimuli. In this situation, the dF/dt or dR/dt terms averaged in time over a whole stimulation cycle are zero.
Thus it appears plausible that the influence of kinetics of the probes on the traces shown in Fig 2 in the main text and on the steady-state values of the $[Ca^{2+}]$ in the three compartments used to validate the model is small.

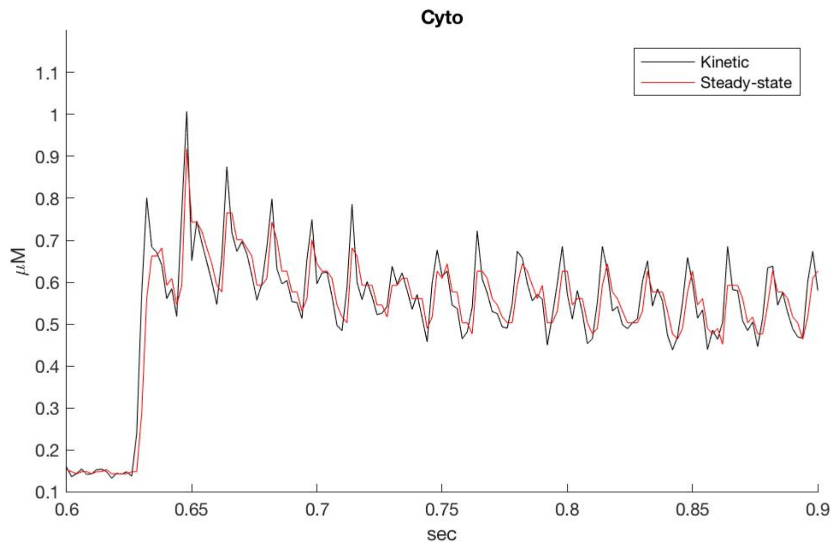

S8 Fig. Kinetic term on the cytosolic $[Ca^{2+}]$. Comparison of the $[Ca^{2+}]_{cyto}$ obtained from a representative Fura-2 recording obtained during 60 Hz stimulation with (black trace) or without (red trace) the kinetic term.

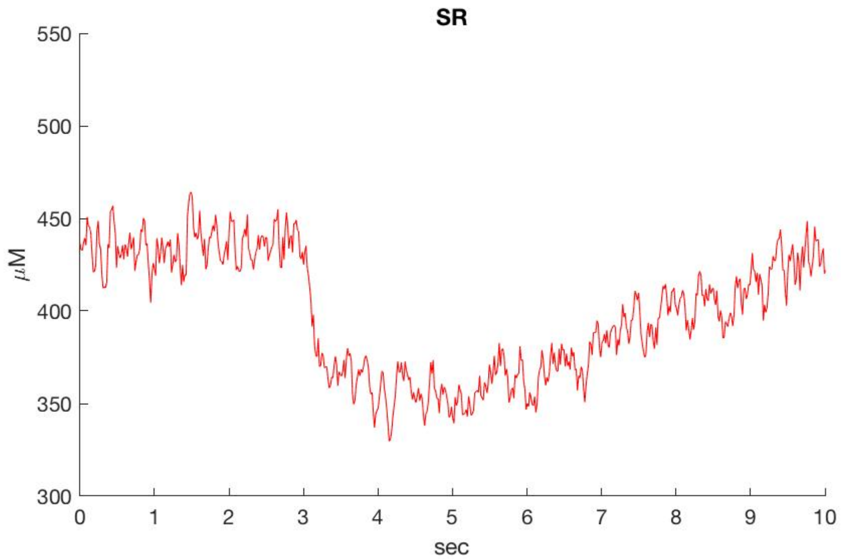

S9 Fig. Kinetic term on the SR $[Ca^{2+}]$. Comparison of the $[Ca^{2+}]_{SR}$ obtained from a representative D1ER recording obtained during 60 Hz stimulation for approximately 2 s with (black trace) or without (red trace) the kinetic term. The two traces are virtually superimposed (the kinetic term accounts for less than 0.03 μM).

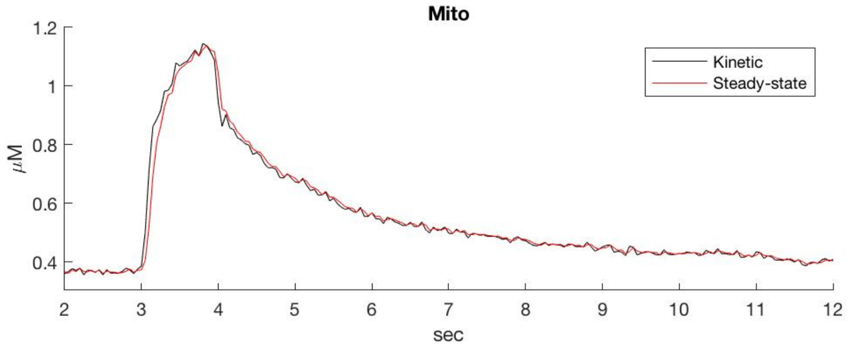

S10 Fig. Kinetic term in mitochondrial [Ca^{2+}]. Comparison of the $[Ca^{2+}]_{mito}$ obtained from a representative 4mtD3cpv recording at 60 Hz for approximately 1 s with (black trace) or without (red trace) the kinetic term for k_{off}=7.6 s^{-1}.

Table S3: Calibration values used for the conversion from fluorescence ratio (F) of Fura-2 acetoxymethyl ester in cytosolic space, or the Förster resonant energy transfer (FRET) ratio (R) of genetically targeted indicators (Cameleon) directed either to the SR (D1ER) or to the mitochondria (4mtD3cpv). For further information see [1] and [2].

.

	F_{min} or R_{min}	F_{max} or R_{max}	K'_d (nM)	k_{off} (s^{-1})	β	n
Cytosol	0.6	2.1	406	217	2.8	-
SR	1.58	2.4	300 10^3	256	1.5	1.67
Mitochondrion	1.53	5.83	4560	7.6	6	0.74

References:

1. Canato M, Scorzeto M, Giacomello M, Protasi F, Reggiani C, Stienen GJM. Massive alterations of sarcoplasmic reticulum free calcium in skeletal muscle fibers lacking calsequestrin revealed by a genetically encoded probe. Proc Natl Acad Sci. 2010;107: 22326–22331. doi:10.1073/pnas.1009168108

2. Scorzeto M, Giacomello M, Toniolo L, Canato M, Blaauw B, Paolini C, et al. Mitochondrial Ca2+-Handling in Fast Skeletal Muscle Fibers from Wild Type and Calsequestrin-Null Mice. Kanzaki M, editor. PLoS ONE. 2013;8: e74919. doi:10.1371/journal.pone.0074919

3. Klein MG, Simon BJ, Szucs G, Schneider MF. Simultaneous recording of calcium transients in skeletal muscle using high- and low-affinity calcium indicators. Biophys J. 1988;53: 971–988.

4. Sztretye M, Yi J, Figueroa L, Zhou J, Royer L, Allen P, et al. Measurement of RyR permeability reveals a role of calsequestrin in termination of SR Ca2+ release in skeletal muscle. J Gen Physiol. 2011;138: 231–247.

5. Palmer AE, Jin C, Reed JC, Tsien RY. Bcl-2-mediated alterations in endoplasmic reticulum Ca2+ analyzed with an improved genetically encoded fluorescent sensor. Proc Natl Acad Sci. 2004;101: 17404–17409. doi:10.1073/pnas.0408030101
