# Supplementary material for: A 3D diffusional-compartmental model of the calcium dynamics in cytosol, sarcoplasmic reticulum and mitochondria of murine skeletal muscle fibers
Source: PLoS One. 2018 Jul 26;13(7):e0201050. doi: 10.1371/journal.pone.0201050 (PMC6062086; doi:10.1371/journal.pone.0201050)
Supplement: S6 File — Time course of the variations in free [Ca2+] for the three compartments at different stimulation rates in WT fibers and CSQ-KO fibers with 10% and 20% of total calcium bound accounted by secondary buffer LB. (PDF) [file pone.0201050.s009.pdf]

S6 File

Time course of the variations in free [Ca^{2+}] for the three compartments at different stimulation rates in WT fibers and CSQ-KO fibers with 10% and 20% of total calcium bound accounted by secondary buffer LB

S11, S12 and S13 Figs. show the simulated oscillations of free calcium concentration in the three compartments (cytosol, SR and mitochondria) for stimulation trains of different duration and frequency: 2, 5, 10 and 15 seconds for 60, 20, 5 and 1 Hz respectively. Figures are for WT model (S11 Fig.), CSQ-KO model when the relative amount of calcium bound to LB is limited to 10% of the calcium bound to CSQ (S12 Fig.), and CSQ-KO model when this amount is increased to 20% (S13 Fig.). Notably, the mitochondrion calcium fails to reach the steady state before the end of the stimulation time, contrary to the experimental observations in the 10% case (S12 Fig.). This further decreases the simulated accumulation of Ca^{2+} inside mitochondrion. The situation is opposite in the 20% case, where the $[Ca^{2+}]_{cyto}$ is close to the experimental data, but the $[Ca^{2+}]_{mito}$ is higher than experimental values (see S13 Fig.).

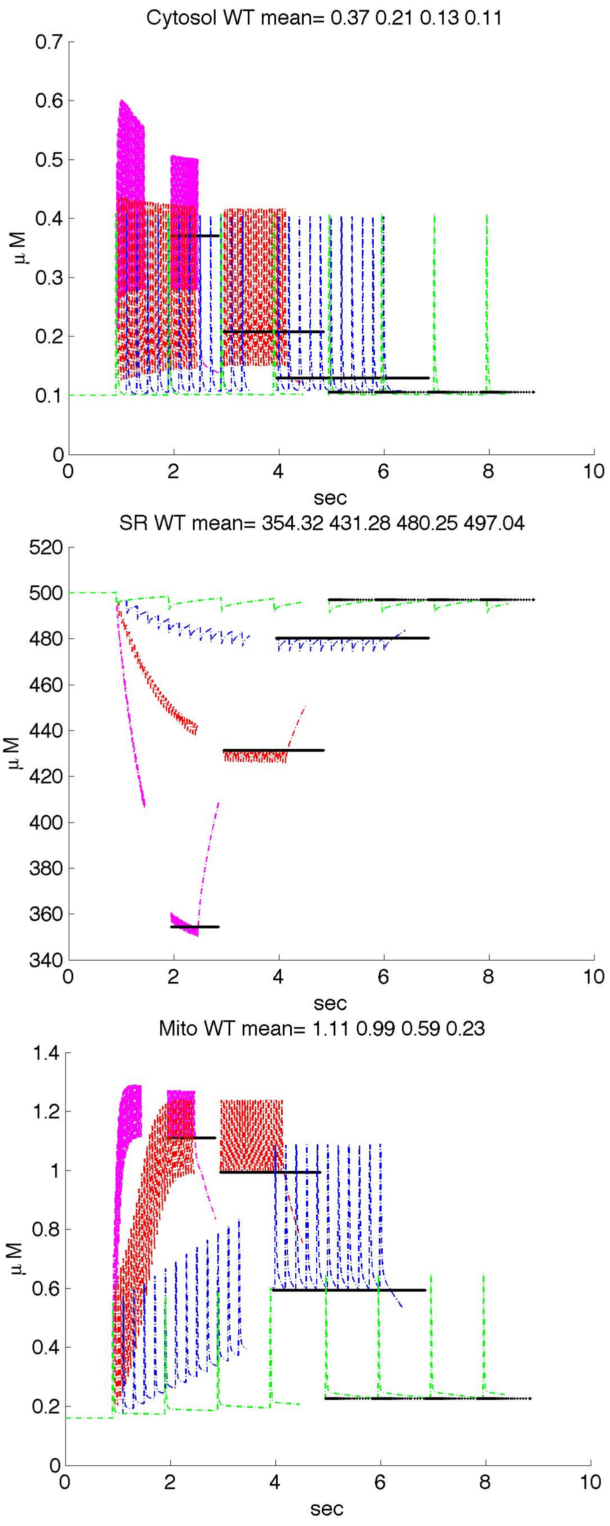

S11 Fig. Kinetics of the [Ca^{2+}] transients in the three compartments (cytosol, SR and mitochondria) of WT fibers for trains of stimulation at 1, 5,20 and 60 Hz. Upper, middle and lower panel shows the calcium concentrations predicted by the model for respectively cytosol, SR and mitochondria during trains of stimulation at 1 Hz (green) with duration 15 s, at 5 Hz (blue) with duration 10 s, at 20 Hz (red) with duration 5 s and at 60 Hz (magenta) with duration 2 s. Only the initial part and the final part of each train are shown. Black lines represent the average values in the cytosol compartment, the upper values in the SR compartment and lower values in the mitochondrial compartment.

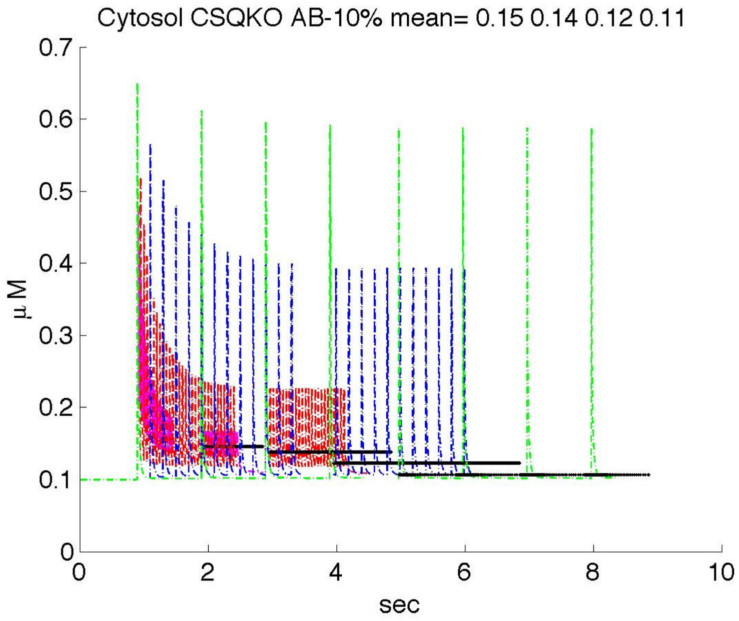
Cytosol CSQKO AB-10% mean= 0.15 0.14 0.12 0.11
μ M
sec

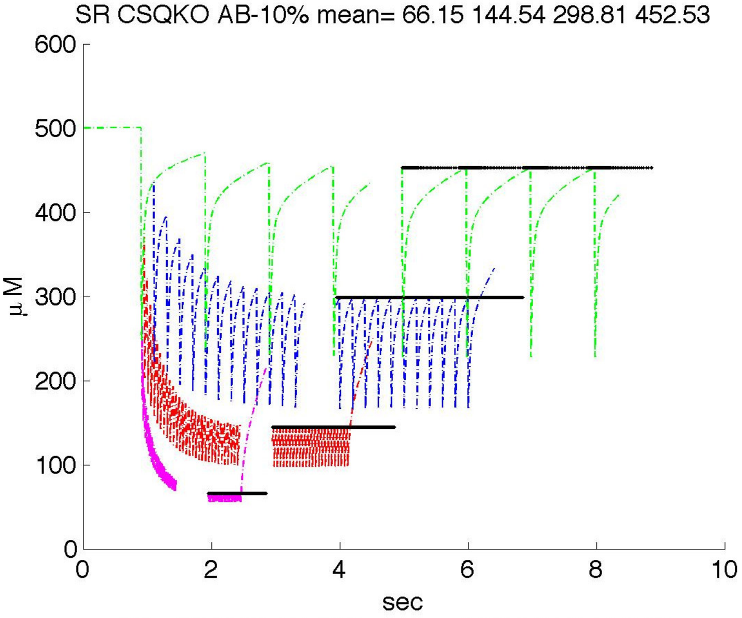
SR CSQKO AB-10% mean= 66.15 144.54 298.81 452.53
μ M
sec

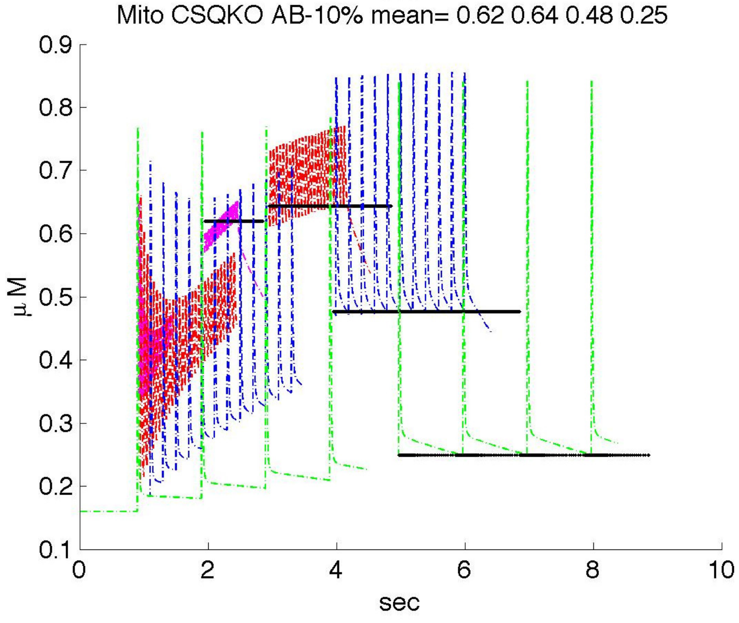
Mito CSQKO AB-10% mean= 0.62 0.64 0.48 0.25
μ M
sec

S12 Fig. Analysis when LB accounts for 10% of total calcium bound. Kinetics of the [Ca^{2+}] transients in the three compartments of CSQ-KO fibers when LB accounts for 10% of total calcium bound, for trains of stimulation at 1, 5, 20 and 60 Hz. Patterns of stimulation and color code as in S11 Fig.

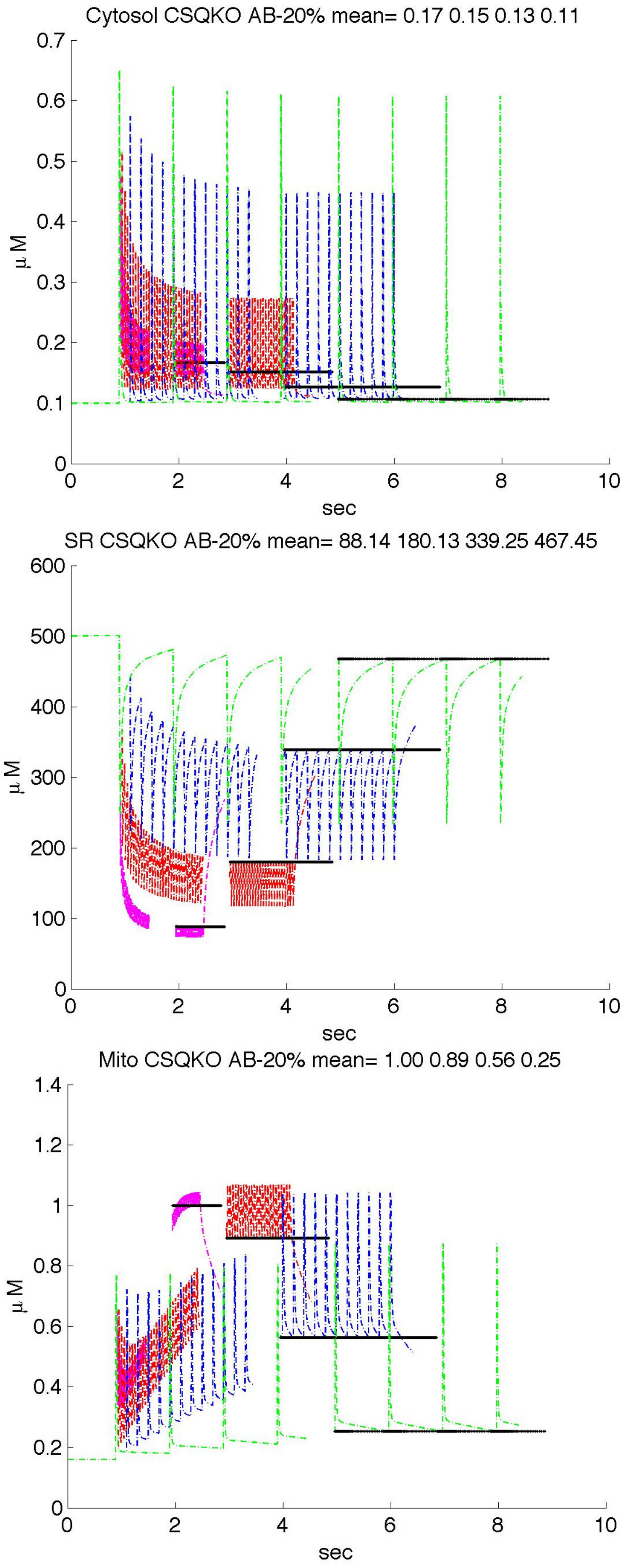
Cytosol CSQKO AB-20% mean= 0.17 0.15 0.13 0.11
μM
sec
SR CSQKO AB-20% mean= 88.14 180.13 339.25 467.45
μM
sec
Mito CSQKO AB-20% mean= 1.00 0.89 0.56 0.25
μM
sec

Fig S13. Analysis when LB accounts for 20% of total calcium bound. Kinetics of the [Ca^{2+}] transients in the three compartments of CSQ-KO fibers when LB accounts for 20% of total calcium bound, for trains of stimulation at 1, 5, 20 and 60 Hz. Patterns of stimulation and color code as in S11 Fig.
